# Comprehensive Evaluation of Quality Indicators: Analyzing the Dutch Breast Cancer Audit

**DOI:** 10.34172/ijhpm.8943

**Published:** 2025-10-01

**Authors:** Elfi M. Verheul, Margrietha van der Linde, Hester F. Lingsma, Elvira Vos, Sabine Siesling, Linetta B. Koppert, NBCA Consortium

**Affiliations:** ^1^Center for Medical Decision Making, Department of Public Health, Erasmus University Medical Center, Rotterdam, The Netherlands.; ^2^Dutch Institute for Clinical Auditing, Leiden, The Netherlands.; ^3^Department of Surgery, Rhode Island Hospital, Warren Alpert Medical School of Brown University, Providence, RI, USA.; ^4^Department of Research, Netherlands Comprehensive Cancer Organization (IKNL), Utrecht, The Netherlands.; ^5^Department of Health Technology and Services Research, Technical Medical Centre, University of Twente, Enschede, The Netherlands.; ^6^Department of Surgery, Erasmus MC Cancer Institute, Rotterdam, The Netherlands.

**Keywords:** Quality Indicators, Quality Indicator Evaluation, Healthcare Quality, Clinical Registries, Cancer Care

## Abstract

**Background:**

Quality indicators (QIs) are widely used to benchmark hospital performance and improve quality of care but are often based on expert opinion rather than data-driven assessment. This study aims to evaluate QIs, using a framework that assesses four criteria: Feasibility, discriminative ability, validity, and reliability.

**Methods:**

We used data from the Dutch breast cancer registry (NABON Breast Cancer Audit, NBCA) and included all surgically treated breast cancer patients in the Netherlands between 2021-2023. Eighteen QIs were evaluated. Feasibility was determined by QI numerator completeness, with >90% data availability considered feasible. Discriminative ability was assessed by between-hospital variation in QI scores, where an interquartile range (IQR) >10% indicated good discrimination. Validity was evaluated by the impact of case-mix adjustment and considered low when (pseudo-) R^2^ <0.10. The (pseudo-)R^2^ reflects the proportion of variance in QI scores explained by all case-mix variables in a regression model. Reliability was assessed by rankability, the proportion of between-hospital variation not due to chance and, therefore, explainable by quality of care. Rankability >75% was considered high.

**Results:**

After exclusion of one QI with feasibility <1%, feasibility ranged from 80.2%-100%, and 15 QIs were feasible. Overall, the IQR ranged from 1 to 36, with 8 QIs having an IQR higher than 10, indicating good discriminative ability. The (pseudo-)R^2^ ranged from 0.01-0.53, with 11 QIs showing low case-mix impact. Rankability ranged from 0-69%, with none of the QIs having a high rankability. None of the QIs met all preset criteria, but six QIs met at least three out of four criteria.

**Conclusion:**

The QIs employed by the Dutch breast cancer registry fulfilled most criteria, but rankability is a concern and requires specific attention, especially for public reporting. Our results show the importance of considering feasibility, discriminative ability, validity, and reliability when evaluating QIs, and these should also be taken into account when developing new QIs.

## Introduction

**Key Messages**

**Implications for policy makers**

- Evaluating quality indicators (QIs) used for provider comparisons is essential to avoid harm caused by steering decisions based on invalid or unreliable data, especially in publicly reporting of data, which has become an increasing concern.
- For public reporting purposes, it is essential to report QI scores of individual hospitals over multiple years or at another aggregate level to achieve higher rankability.
- New QIs should be developed with the criteria feasibility, discriminative ability, validity, and reliability in mind, to avoid creating invalid QIs based solely on expert opinion.
- We provide a practical guide for a data-driven evaluation of QIs to assess feasibility, discriminative ability, validity, and reliability, which could be applied in other quality registries.

**Implications for the public**

As quality of care is difficult to measure, quality indicators (QIs) aim to provide signals of quality. Especially when publicly reported, it is important that individual hospitals scores are valid and reliable to prevent unnecessary harm. Valid and reliable QIs guide healthcare providers improving care, assist patients in making informed choices about their care providers’ quality and support designing pay-for-performance strategies that incentivise high-quality care. Using a framework of the European Observatory on Health Systems and Policies, we evaluated all QIs for breast cancer care in the Netherlands. We found that while most criteria are met, reliability remains a concern. For more reliable public reporting, we recommend reporting QI scores over multiple years or at an aggregate level to improve reliability. Our findings directly impact the Dutch breast cancer quality registry and could also influence other quality registries if they adopt our approach, as outlined in our practical guide.

The increasing interest in identifying and addressing variation in quality of care is supported by quality registries,^1^ which aim to assess and monitor healthcare performance using outcome, process, and structure indicators.^2^ These Quality indicators (QIs) have multiple applications, including monitoring and benchmarking. Internally, organizations such as hospitals use the QIs to improve their own practices, while benchmarking involves comparing hospitals’ performance with that of others or national standards. Benchmarking sometimes includes public reporting, where QIs are made publicly available to support patient decision-making, contracting of care by health insurers, and evaluations by regulators.^3,4^

 For benchmarking, QIs must accurately measure quality of care, requiring them to be both valid and reliable.^5,6^ If QIs are valid and reliable, observed variation may actually represent differences in quality. Meaningful benchmarking also depends on adequate data quality; however, achieving this quality also creates an administrative burden, potentially undermining the feasibility. Additionally, to clearly identify areas for improvement, indicators must have sufficient discriminative ability.

 In 2011, the multidisciplinary NABON Breast Cancer Audit (NBCA) was developed to measure the quality of care in surgically treated Dutch breast cancer patients. Initially the set consisted of thirty-two Qis,^7^ and it evolved over time. In the context of the NBCA, there is an annual assessment of the QI set based on expert opinion by the scientific committee.^7^ New QIs are introduced based on new insights and clinical guideline revisions, while existing indicators are removed from the set when they are presumed to no longer meet the criteria of feasibility, relevance, validity, and reliability.^1,7,8^

 Some of these criteria have been (partly) assessed empirically in the NBCA. Feasibility has proven to be good, as all hospitals are required to provide data to the NBCA.^9^ Validity and reliability of six QIs were evaluated during 2011-2016 by Vos et al,^5^ which showed some deficiencies in both criteria. Given that the QI set used in 2024 consists of 19 partly new QIs, the majority have not undergone any assessment. The aim of this study is to perform a comprehensive quantitative evaluation of the current QI set of the NBCA using a framework designed for the assessment of QIs.^10^

## Methods

###  Data

 We used data from the NBCA. This Dutch national quality registry includes all patients surgically treated for breast cancer in the Netherlands, including ductal carcinoma in situ (DCIS), invasive carcinoma, Paget’s disease and inflammatory carcinoma. Hospitals can either register the data themselves (20% of the hospitals) or make use of the Netherlands Cancer Registry hosted by the Netherlands Comprehensive Cancer Organisation (IKNL). We included all patients that underwent surgery during the years 2021-2023.

###  Quality Indicators 

 We considered 19 indicators for evaluation (Table 1). The QI set of the Dutch breast cancer quality registry of 2024 consists of 18 indicators, categorized according to Donabedian’s framework: One structure indicator, four outcome indicators, and 14 process indicators.^2^ We added the QI “MRI (magnetic resonance imaging)for lobular breast cancer” which was included in the set of 2023, but removed in 2024 after its objective was met.^11^ The structure indicator (QI-1) was excluded from the data-driven assessment due to methodological limitations in this format. All other QIs were included. Nine QIs are applied to different subgroups, related to the type of surgery or treatment trajectory.

**Table 1:** Description of Quality Indicators Selected for Evaluation

**QI**	**Type of Indicator**	**Definition**	**Development Year**^[1]^	**Currently Publicly Reported**
QI-1	Structure	**BC team** QI1a: Number of newly diagnosed patients with invasive BC or DCIS who underwent surgical treatment QI1b: Number of certified medical oncologists treating BC QI1c: Number of certified oncologic surgeons treating BC QI3d: Number of plastic surgeons performing reconstruction surgery QI3e: Number of patients who have their own case manager, according to the SONCOS^[2]^ definition, and whether this is registered in the patient's file QI3f: Is there documented collaboration with, or involvement in, the multidisciplinary team of a clinical genetics department? QI3g: Is the collection of family medical history, the assessment of referral criteria, the availability of genetic testing, and the provision of urgent counselling and DNA diagnostics structurally integrated into the care pathway?	a,b,c,d: 2019 e: 2020 f,g: 2024	Yes
QI-2	Process	**Breast contour preserving treatment** *Numerators*: Number of patients who underwent breast contour preserving treatment after: QI2a: Surgery (b+c+d) QI2b: Breast-conserving surgery including re-lumpectomies without neoadjuvant chemotherapy^[3]^ QI2c: Breast-conserving surgery following neoadjuvant systemic^[3]^ therapy QI2d: Mastectomy with immediate reconstruction (autologous and/or prothesis) *Denominator*: Number of surgically treated patients with non-metastasized BC^[2]^	2016	Yes
QI-3	Process	**Immediate breast reconstruction in invasive disease** ^[4]^ *Numerator*: Number of patients who underwent immediate reconstruction after mastectomy for invasive non-metastasized BC^[2]^, where reconstruction is performed by a plastic surgeon: QI3a: Immediate reconstruction, total (b+c+d+e) QI3b: Immediate reconstruction with prostheses QI3c: Immediate autologous reconstruction QI3d: Immediate reconstruction with prostheses and autologous tissue QI3e: Immediate reconstruction, other *Denominator*: Number of patients undergoing mastectomy for invasive non-metastasized BC^[4]^	2012	Yes
QI-4	Process	**Immediate breast reconstruction in DCIS** *Numerator*: Number of patients who underwent immediate reconstruction after mastectomy for DCIS, where reconstruction is performed by a plastic surgeon: QI4a: Immediate reconstruction, total (b+c+d+e) QI4b: Immediate reconstruction with prostheses QI4c: Immediate autologous reconstruction QI4d: Immediate reconstruction with both prostheses and autologous tissue QI4e: Immediate reconstruction, other *Denominator*: Number of patients undergoing mastectomy for DCIS	2012	Yes
QI-5	Process	**Seen by radiation oncologist prior to neoadjuvant chemotherapy** *Numerator*: Number of patients seen by the radiotherapist within 28 days of starting treatment *Denominator*: Number of patients with invasive BC treated with neoadjuvant chemotherapy^[3]^, surgery, and postoperative radiotherapy	2011	Yes
QI-6	Process	**Neoadjuvant systemic therapy** ^[3]^ **for patients with triple negative and HER2 positive BC** *Numerator*: Number of patients receiving neoadjuvant systemic therapy^[3]^ *Denominator*: Number of patients under 70 years old with cT2/3/4 any N M0 triple-negative or HER2/Neu positive BC^[4]^	2020	Yes
QI-7	Process	**Time between diagnosis of IBC and primary treatment** Median time in calendar days between date of biopsy where diagnosis is established and the start of: QI7a: Any treatment (b+c+d) QI7b: Neoadjuvant chemotherapy^[3]^ QI7c: First surgery (excluding direct reconstruction) QI7d: First surgery with direct reconstruction	2011 (2011- 2017 in binary form: <5 weeks yes/no)	Yes
QI-8	Process	**PROMs response** *Numerator*: Completing PROM^[5]^ questionnaires at T0 (before start treatment) and at T1 (1 year after start treatment) *Denominator*: Number of patients registered in the NBCA	2019	Yes
QI-9	Process	**MRI-mamma prior to neoadjuvant chemotherapy** *Numerator*: Number of patients who received breast MRI prior to the start of neoadjuvant chemotherapy^[3]^ *Denominator*: Number of patients with primary invasive non-metastasized BC treated with neoadjuvant chemotherapy	2012	Yes
QI-10	Process	**Radiotherapy for locally advanced BC requiring mastectomy** *Numerator*: Number of patients receiving radiotherapy after mastectomy *Denominator*: Number of patients with primary invasive locally advanced non-metastasized BC^[4]^ without distant metastases (Dutch guideline: cT4, pT4, pT3N1 or ≥pN2) who underwent a mastectomy	2011	No
QI-11	Outcome	**Irradical resection in primary breast-conserving surgery for invasive BC** *Numerator*: Number of patients with more than focally positive margins^[6]^ *Denominator*: Number of patients who underwent first breast-conserving surgery (without neoadjuvant therapy^[3]^) for primary invasive non-metastasized BC^[4]^	2011	No
QI-12	Outcome	**Irradical resection in primary breast-conserving surgery for DCIS** *Numerator*: Number of patients with positive margins *Denominator*: Number of patients who underwent a first breast-conserving surgery for DCIS	2011	No
QI-13	Process	**Time from last surgery to start adjuvant therapy** Median time in calendar days between the last surgery and: QI13a: Adjuvant treatment QI13b: Adjuvant chemotherapy QI13c: Adjuvant radiotherapy *Denominator*: Number of surgical treated patients with primary invasive non-metastasized BC and/or DCIS	2011 (2011- 2017 in binary form: <5 weeks yes/no)	No
QI-14	Process	**Trial participation** *Numerator*: Number of patients who participated in a registered clinical trial *Denominator*: All surgically treated patients with invasive BC or DCIS	2020	No
QI-15	Outcome	**Complicated course after surgery** *Numerator*: Number of patients with a complicated course^[7]^ *Denominator*: Number of patients with invasive BC or DCIS receiving: QI15a: Lumpectomy QI15b: Mastectomy without immediate reconstruction QI15c: Mastectomy with immediate reconstruction	2020	No
QI-16	Outcome	**Complicated course after chemotherapy** *Numerator*: Number of patients with a complicated^[8]^ course after QI16a: Chemotherapy, total (b+c) QI16b: Neoadjuvant chemotherapy QI16c: Adjuvant chemotherapy *Denominator*: Number of patients with invasive non-metastasized BC treated with chemotherapy and underwent surgery	2020	No
QI-17	Process	**Collaboration of surgeon and plastic surgeon for invasive BC** *Numerator*: Number of patients receiving surgery by a general surgeon and plastic surgeon together *Denominator*: Number of patients diagnosed with invasive non-metastasized BC undergoing primary breast-conserving surgery	2022	No
QI-18	Process	**Collaboration of surgeon and plastic surgeon for DCIS** *Numerator*: Number of patients receiving surgery by a general surgeon and plastic surgeon together *Denominator*: Number of patients diagnosed with DCIS undergoing primary breast-conserving surgery	2022	No
QI-19	Process	**MRI for lobular breast cancer** *Numerator*: Number of patients with lobular BC receiving an MRI QI19a: Prior to surgery, total (b+c) QI19b: Prior to breast conserving surgery QI19c: Prior to mastectomy *Denominator*: Number of patients diagnosed with lobular non-metastasized BC undergoing surgery (*a*), primary breast-conserving surgery (*b*) or mastectomy (c*)*	2017	No^[9]^

Abbreviations: QI, quality indicator; BC, breast cancer; DCIS, ductal carcinoma in situ; IBC, invasive breast cancer; NBCA, NABON Breast Cancer Audit; PROM, patient reported outcome measurement; MRI, magnetic resonance imaging; NA, not applicable. ^[1]^For indicators developed before 2019, there was no distinction between internal and external use of QIs, this implicates that QIs were made publicly available directly with their development. It is possible that QIs the transparency of some QIs have been reverted. ^[2]^SONCOS is the Dutch multidisciplinary oncology platform aimed at describing the standards of quality of oncologic care that must be met. These standards are established by oncological professionals.^9^ ^[3]^Neoadjuvant systemic therapy is defined as neoadjuvant chemotherapy and/or HER2 blockade. ^[4]^Invasive BC can be with or without a DCIS component. ^[5]^PROMs consisting of: EORTC-QLQ-C30, and/or EORTC-QLQ-BR23, and/or BREAST-Q. ^[6]^More than focally positive margins is defined as tumour touching the inked margin over a length of 4 mm or more. ^[7]^A complicated course is defined as grade 3 and higher according to the Clavien-Dindo classification.^12^ ^[8]^A complicated course is defined as premature discontinuation or clinical admission due to adverse effects of chemotherapy. ^[9]^This QI was publicly available from 2020-2023 but removed from the set in 2024.

 QI scores were calculated based on numerator and denominator definitions per hospital (Table 1). Binary process indicators (ie, adherence scores) were calculated by dividing the number of patients who adhered to the QI for each individual hospital (numerator) by the number of patients per hospital to whom the QI was applicable (denominator). Continuous process indicators QI-7 and QI-13 were calculated as the median time in calendar days per year. Outcome indicators were calculated by dividing the number of events (ie, the occurrence of the specific QI) for each hospital (numerator) by the total number of patients per hospital who were eligible to be included in the indicator (denominator).

###  Framework

 The European Observatory on Health Systems and Policies summarised several frameworks to assess the quality of indicators, to standardise the evaluation (Supplementary file 1, Figure S1).^13^ The authors emphasise that based on the goal of the QI and the specific setting the relative importance of each of the criteria may differ. For example, when QIs are publicly available, the criteria validity and reliability are more important to ensure fair hospital comparisons.^5,14^ For the evaluation of the QIs in this study, we selected the criteria that were both relevant for between-hospital comparisons and were quantitatively assessable. These criteria were feasibility, discriminative ability, validity, and reliability, and were evaluated for all the 18 selected QIs.

###  Statistical Analysis

 We presented descriptive statistics as count and percentages or mean with standard deviation (SD), separately for calendar year 2023 and for combined years 2021-2023. Missing baseline characteristics were imputed using single imputation, assuming values were missing at random. We fitted the regression imputation model with all outcomes and potential predictors. Missing values in the QI numerator were recoded to zero, as this is common practice in the Netherlands. Depending on the QI definition, this “numerator to zero” approach can positively or negatively impact the QI score. For example, for the QI complicated course after surgery (QI-15), a lower score indicates better performance. On the contrary, for MRI-mamma prior to neoadjuvant chemotherapy (QI-9), a lower score might be interpreted as worse performance. We used this approach for the evaluation of the criteria discriminative ability, influence of case-mix and rankability. We evaluated all criteria on the most recent calendar year (2023) to align as closely as possible with the way indicators are used in practice, except for the influence of case-mix which was assessed over the years 2021-2023. Additionally, we performed a sensitivity analysis for the QI with the lowest feasibility (QI-5) by imputing outcomes using a logistic regression model for single imputation.

####  Feasibility

 Feasibility encompasses data quality and availability.^15^ We quantified feasibility by calculating the completeness of the QI numerator, having already defined the denominator population. To determine feasibility, we used and extended thresholds set by Huijben et al,^16^ classifying data completeness of the numerator above 90% as good, between 70%-90% as moderate, and below 70% as poor. If feasibility was <25%, the QI was excluded for evaluation on remaining criteria.

####  Discriminative Ability: Between-Hospital Variation

 Discriminative ability, further denoted as between-hospital variation, means the ability of a QI to distinguish in hospital performance. If there is only little hospital variation, there may be small improvement possibilities, while wide hospital variation offers more room for improvement. We quantified between-hospital variation as the median and interquartile range (IQR) of the QI score. Discriminative ability was categorised as poor (IQR < 5), moderate (IQR 5-10), and good (IQR > 10).

####  Validity: Influence of Case-Mix Adjustment

 Although validity is a broad concept, in this study we only assessed the impact of adjustment for baseline patient and tumour characteristics (ie, case-mix). In the context of between-hospital comparisons little impact of case-mix adjustment is favourable, as with high case-mix influence observed between-hospital variation in the QI score is more likely due to differences in underlying patient population rather than quality of care. We combined three years of data (2021-2023) to yield more robust results, and fitted a case-mix model for each QI separately. We identified potential case-mix variables through literature and expert opinion, which were age, sex, body mass index (BMI), smoking, multifocality, cTNM-stage, histology, differentiation grade, and receptor status.^5^ For the continuous variable age, we checked for non-linearity of the association between predictor and outcome. We assessed model performance using Nagelkerke’s pseudo-R^2^ for the binary QIs and the R^2^ for the continuous QIs. The (pseudo-)R^2^ quantifies the extent to which the variance in a QI score is explained by the predictors in the model.^17^ The closer the (pseudo-)R^2^ is to 1, the better the model can explain why patients have different outcomes. We considered a (pseudo-)R^2^ ≥ 0.25 as significant, a (pseudo-)R^2^ between 0.10-0.25 as moderate, and a (pseudo-)R^2^ lower than 0.10 as minimal case-mix influence.

 When patient populations are comparable across hospitals, the influence of adjustment on hospital comparisons is likely to be minimal, even if the case-mix variables have a large impact on the QI. Therefore, we further assessed the effect of case-mix adjustment on hospital comparisons using the observed/expected (O/E)-ratio. This ratio is determined by dividing the observed by the expected number of events per hospital. An O/E-ratio exceeding 1 indicates more events than expected, while below 1 indicates fewer. We estimated both unadjusted and case-mix adjusted O/E-ratios. The observed number of events for each ratio was the individual hospital QI score. For the unadjusted O/E ratio, the expected number of events was calculated as the mean QI score across all hospitals for binary QIs and the mean number of days across all hospitals for continuous QIs. For the case-mix adjusted O/E ratio, the expected number of events was determined as the predicted probability for each hospital for binary QIs and the predicted number of days for each hospital for continuous QIs, with both estimates derived from the case-mix adjustment model. We visualised the O/E-ratios for each hospital in a scatterplot, with the y-axis showing unadjusted and the x-axis the case-mix adjusted O/E-ratio. The total deviation from the diagonal was quantified with the root mean squared error (RMSE), which is the square root of the average squared deviance per hospital. A lower RMSE indicates a smaller effect of case-mix adjustment on hospital comparisons. If the hospitals are exactly on the diagonal, there is no effect of case-mix adjustment (RMSE = 0).

####  Reliability: Rankability

 Reliability is related to the ability of QIs to meaningfully distinguish hospitals. We operationalized this with rankability, which reflects the proportion of variation that is not due to chance.^18^ For example, a rankability of 60% indicates that 60% of theobserved differences are differences not caused by chance, but potentially by quality of care. Rankability is calculated using formula [1], in which *ρ* denotes rankability, τ^2^ denotes the variance of a random effects model where hospital is added as a random intercept (ie, between-hospital variation), and *S*_i_denotes the standard error of the estimated hospital effect for hospital *i*. The standard error reflects the precision with which the hospital fixed effects are estimated and is highly impacted by the total number of patients treated in that hospital. Rankability is categorised as low(<50%), moderate (50%-75%), or high (>75%).^5^


[1] $$\rho =\frac{\tau^{2}}{\tau^{2}+median(s_{i}^{2})}$$

For the calculation of the rankability, we excluded hospitals that treated fewer than two patients per year, for technical reasons. Additionally, in some instances we used a simplification of the full case-mix model leaving out variables with no events (Supplementary file 1, Table S1). We performed sensitivity analyses by calculating rankability over three calendar years (2021-2023) to determine if this approach enhances rankability.

 After the analysis, we evaluated which of the QIs met all the criteria to determine their overall performance.

###  Practical Guide

 We developed a practical guide, including R codes, which is applicable for QI evaluation of other registries. This guide can be found in Supplementary file 2. All analyses were performed using RStudio, version 4.2.3.

## Results

###  Descriptive Statistics

 In 2023, 15 301 patients with invasive breast cancer (IBC) and 1953 patients with DCIS were surgically treated across 71 hospitals in the Netherlands (Table 2). Most patients underwent breast conserving surgery (73%) in comparison with mastectomy (27%). Around one-third of the patients who underwent a mastectomy also received an immediate reconstruction (IBC: 26%, DCIS: 40%). A quarter of the IBC patients underwent neoadjuvant chemotherapy (26%). Patients with DCIS less frequently received radiotherapy (45% vs. 69%) and were not treated with systemic therapy. The distribution of patient- tumour and treatment characteristics in 2023 was similar to that of the 2021-2023 cohort.

**Table 2:** Baseline Characteristics of Included Patients (Year 2023)

	**2023**				**2021-2023**			
	**IBC (N = 15** **301)**		**DCIS (N = 1953)**		**IBC (N = 46** **187)**		**DCIS (N = 5787)**	
	**Total** **N (%)**	**Missing** **N**	**Total** **N (%)**	**Missing** **N**	**Total** **N (%)**	**Missing** **N**	**Total** **N (%)**	**Missing** **N**
** Patient Characteristics**								
Age, mean (SD)	63 (13)	0	62 (11)	0	62 (13)	10	61 (11)	1
BMI (SD)	27 (5)	2608	26 (5)	240	27 (5)	5987	26 (5)	688
Sex		0		0		0		0
Female	15 196 (99%)		1941 (99%)		45 864 (99%)		5742 (99%)	
Male	105 (1%)		12 (1%)		323 (1%)		45 (1%)	
Smoking		4192		568		12 090		1678
No or quitted	9170 (83%)		1199 (87%)		28 363 (83%)		3534 (86%)	
Yes	1939 (17%)		186 (13%)		5734 (17%)		575 (14%)	
**Tumour Characteristics**								
T-stage		164				620		
cT1	8331 (55%)				25 154 (55%)			
cT2	5141 (34%)				15 307 (34%)			
cT3/4	1250 (8%)				3872 (8%)			
cTis	415 (3%)				1234 (3%)			
N-stage		107				385		
cN0	12 534 (83%)				37 733 (82%)			
cN+	2656 (17%)				8069 (18%)			
Receptor status		0				0		
HR+, Her2-	11 231 (73%)				34 388 (74%)			
Her2+	1792 (12%)				5548 (12%)			
Triple-	2278 (15%)				6251 (14%)			
Differentiation grade		2202		170		4276		507
1	3146 (24%)		274 (15%)		9711 (23%)		801 (15%)	
2	7167 (55%)		671 (38%)		22 570 (54%)		1975 (38%)	
3	2786 (21%)		829 (47%)		9630 (23%)		2504 (47%)	
Histology		1851				3359		
No special type	10 399 (77%)				33 074 (77%)			
Lobular	1784 (13%)				5668 (13%)			
Other	1327 (10%)				4086 (10%)			
Multifocality		2626		150		5194		363
Unifocal	10 362 (82%)		1710 (95%)		33 378 (81%)		5154 (95%)	
Multifocal	2313 (18%)		93 (5%)		7615 (19%)		270 (5%)	
**Treatment Characteristics**								
Type of surgery		1652^a^		43		2608		170
Lumpectomy	9799 (72%)		1388 (73%)		30 765 (71%)		4077 (73%)	
Mastectomy	3850 (28%)		522 (27%)		12 814 (29%)		1540 (27%)	
Immediate reconstruction post-mastectomy^b^	1056 (26%)	5	223 (40%)	0	3459 (27%)	6	660 (43%)	0
Neoadjuvant chemotherapy	3971 (26%)	37			12 320 (27%)	50		
Adjuvant chemotherapy	1447 (10%)	230			5571 (12%)			
Radiotherapy	9449 (69%)	1568	875 (45%)	23	31 400 (72%)	2303	2644 (46%)	60
Hormone therapy	1495 (49%)	12 279^c^			16 147 (48%)	12 613		

Abbreviations: IBC, Invasive breast cancer; DCIS, ductal carcinoma in situ; SD, standard deviation; BMI, body mass index. ^a^1429 patients are coded as “intention to receive surgery.” The specific type of surgery is not yet determined, given that they are still receiving neo-adjuvant chemotherapy (the inclusion year is the time of biopsy). ^b^Only patients receiving mastectomy are included in this variable. ^c^High number of missingness due to exclusion of the data-entry set of the Netherlands Comprehensive Cancer Organisation in 2023. The ratio is comparable to that observed in 2022.

###  Feasibility

 For nearly all QIs included the numerators were complete for >90%, and thus were feasible (Table 3). Moderate feasibility was observed for the QIs “seen by radiation oncologists prior to neoadjuvant chemotherapy” (QI-5), and “complications after chemotherapy” (QI-16C). QI-8, patient reported outcome measurements(PROMs) response, showed little feasibility and was consequently excluded from further evaluation.

**Table 3:** Overview of Quality Indicator Performance

**QI**^a^	**No. of Patients**^b^	**Patients Per Hospital (Median, IQR)**	**Feasibility** **% Data Available (Hospital range)**^c^	**Discriminative Ability** **Between Hospital Variation**^d^ **Median (IQR)**	**Case-mix** **(Pseudo)R-squared**^e^	**Reliability** **Rankability%**
**Year(s) of data**	**2023**	**2023**	**2023 **	**2023 **	**2021-2023**	**2023**
QI-2						
QI-2A	13 502	186 (108-250)	99.7 (61-100)	76 (72-80)	0.26	32
QI-2B	13 502	186 (108-250)	99.8 (61-100)	52 (47-56)	0.53	22
QI-2C	13 502	186 (108-250)	99.8 (61-100)	16 (13-19)	0.35	16
QI-2D	13 502	186 (108-250)	99.9 (96-100)	7 (5-11)	0.28	31
QI-3						
QI-3A	3881	54 (34-70)	99.9 (96-100)	23 (17-34)	0.38	37
QI-3B	3881	54 (34-70)	99.9 (96-100)	20 (13-27)	0.33	33
QI-3C	3881	54 (34-70)	99.9 (96-100)	1 (0-3)	0.10	32
QI-3D	3881	54 (34-70)	99.9 (96-100)	0 (0-1)	0.05	16
QI-3E	3881	54 (34-70)	99.9 (96-100)	0 (0-1)	0.09	NA
QI-4						
QI-4A	533	7 (4-10)	100 (100-100)	39 (28-50)	0.36	8
QI-4B	533	7 (4-10)	100 (100-100)	28 (21-40)	0.27	8
QI-4C	533	7 (4-10)	100 (100-100)	0 (0-7)	0.07	NA
QI-4D	533	7 (4-10)	100 (100-100)	0 (0-0)	0.05	NA
QI-4E	533	7 (4-10)	100 (100-100)	0 (0-0)	0.10	NA
QI-5	1812	23 (14-36)	80.2 (0-100)	74 (52-88)	0.02	61
QI-6	1466	19 (10-27)	99.8 (75-100)	94 (92-97)	0.10	0
QI-7						
QI-7A	13 976	190 (110-256)	92.5 (61-100)	29 (23-38)*	0.03	66
QI-7B	3792	47 (28-72)	99.4 (64-100)	28 (22-34)*	0.03	39
QI-7C	9532	134 (78-180)	100 (96-100)	30 (23-39)*	0.04	66
QI-7D	652	9 (4-12)	100 (100-100)	42 (32-55)*	0.02	27
QI-8	15 596		<1	-	-	-
QI-9	2773	35 (20-53)	99.5 (64-100)	94 (91-97)	0.07	69
QI-10	535	7 (4-10)	100 (70-100)	75 (58-90)	0.12	0
QI-11	7528	101 (61-141)	99.4 (95-100)	2 (1-3)	0.06	0
QI-12	1384	18 (11-26)	99.9 (96-100)	17 (12-21)	0.03	15
QI-13						
QI13-A	10 533	134 (88-198)	94.4 (94-100)	40 (34-48)*	0.02	52
QI13-B	575	6 (3-11)	100 (100-100)	35 (28-44)*	0.02	23
QI13-C	9412	121 (76-180)	100 (100-100)	40 (34-48)*	0.02	56
QI-14	15 596	213 (128-285)	92.5 (0-100)	4 (3-7)	0.04	30
QI-15						
QI15-A	15 596	139 (91-210)	93.8 (0-100)	2 (1-3)	0.01	0
QI15-B	11 187	44 (26-60)	94 (0-100)	5 (3-8)	0.01	13
QI15-C	4372	14 (10-19)	91.8 (0-100)	10 (5-16)	0.02	0
QI-16						
QI16-A	3785	47 (30-72)	90.5 (0-100)	28 (23-33)	0.03	24
QI16-B	2773	35 (20-53)	91.1 (0-100)	29 (22-34)	0.03	25
QI16-C	1406	18 (8-26)	87.8 (0-100)	31 (24-38)	0.03	0
QI-17	9516	122 (78-179)	97.8 (0-100)	8 (4-13)	0.10	67
QI-18	1347	18 (10-26)	97.9 (0-100)	8 (4-14)	0.05	37
QI-19						
QI19-A	1408	17 (11-27)	100 (100-100)	84 (74-98)	0.17	0
QI19-B	889	11 (7-18)	100 (100-100)	87 (76-92)	0.14	53
QI19-C	519	7 (4-10)	100 (100-100)	81 (72-86)	0.25	20

Abbreviations: IQR, interquartile range; QI, quality indicator; NA, not applicable. ^*^Number of days. ^a^See Table 1 for exact definitions of all QIs. ^b^The number of patients in denominator. ^c^Hosital range (minimum-maximum) of percentage data available. ^d^This is the QI score expressed as a percentage, except for continuous outcomes QI7 and QI12. For these QIs, the score represents the median value for each hospital, from which the overall median and IQR were calculated. ^e^For continuous outcomes QI7 and QI12 the R-squared is presented instead of the pseudo R-squared. The colours indicate poor (orange), moderate (yellow) and good (green) performance on the selected criteria.

###  Between-Hospital Variation

 Half of the QIs showed an IQR > 10, suggesting notable differences between hospitals (Table 3). Low between-hospital variation was mostly observed in QIs with either high (QI-6, QI-9) or low median scores (QI-3C/D/E, QI-4C/D/E, QI-11, QI-14, QI-15).

###  Case-Mix

 For four QIs, there was significant impact of patient and tumour characteristics on the QI score, reflected in the pseudo-R^2^ > 0.25 (Table 3). Three out of these four QIs are related to surgery, namely breast contour preserving surgery (QI-2), and immediate reconstruction after IBC and DCIS (QI-3, QI-4). The fourth QI substantially affected by case-mix was MRI for lobular BC before mastectomy (QI-19C). Most QIs that have a pseudo-R^2^ exceeding 0.25 also exhibit high RMSE scores (Supplementary file 1, Table S2, Figure S2).

###  Rankability

 None of the QIs showed good rankability (>75%). The highest score was observed for QI-9, MRI-mamma prior to neoadjuvant chemotherapy, which achieved a rankability of 69%. Moderate rankability scores were observed in five QIs (QI-5, QI-7A/C, QI-9, QI-13A, QI-17). Furthermore, all QIs with a low number of patients included in the denominator, or a low number of patients in the nominator showed poor rankability (<50%). For QI-3E and QI-3C/D/E, it was not possible to calculate rankability, as the number of included patients in the numerator was too small for individual hospitals (Supplementary file 1, Table S1). Combining data of 3 years generally resulted in higher rankabilities (Supplementary file 1, Table S3).

###  Performance of Quality Indicators

 None of the QIs met the predefined thresholds on all evaluated criteria. However, six QIs met three out of four criteria. These were time between diagnosis of IBC and primary treatment (QI-7A, QI-7C), radiotherapy for locally advanced BC requiring mastectomy (QI-10), median time last surgery to start any adjuvant therapy (QI-13A), complicated course after mastectomy (QI-15C), complicated course after chemotherapy and after neoadjuvant chemotherapy (QI-16A/B) and collaboration of surgeon and plastic surgeon for DCIS (QI-18). Rankability was observed as the least met criteria, with only (subgroups of) 5 out of 18 QIs having moderate rankability, and the remaining showing poor rankability. All of four outcome indicators showed poor rankability, and three out of four showed moderate to no between-hospital variation. The sensitivity analyses also emphasized the importance of good feasibility, particularly for a reliable benchmark (Supplementary file 3, Figure S3 and Table S4).

## Discussion

 This study aimed to assess all QIs of the Dutch Breast Cancer Quality Registry (NBCA), applying a comprehensive framework that includes criteria feasibility, discriminative ability, validity, and rankability. None of the QIs fulfilled all criteria, but in 6 QIs three out of the four criteria were met. Feasibility was the most met criterion, with 16 out of 18 QIs performing well, while rankability was the least met with only five QIs showing moderate rankability and the remaining demonstrating poor rankability. Across outcome indicators we observed a pattern of both poor to moderate discriminative ability and rankability.

###  Evaluation on Criteria

 Data availability was high for nearly all QIs, likely related to the fact that about 80% of the hospitals use data from the Netherlands Cancer Registry. Furthermore, the collection of QIs over multiple years may further account for the observed high feasibility; for example, QI-4, collected since 2012, currently demonstrates 100% feasibility.^19^ However, for six QIs we observed at least one hospital not providing any data (reflected in feasibility range starting at zero). Three potential explanations may account for this finding. First, five of these QIs were developed after 2020 (three in 2020 and two in 2022), which may suggest limited time for implementation. Second, only one of the six QIs is publicly available; hospitals might prioritize transparent QIs, and therefore, these could theoretically have higher feasibility. Third, variation in the method of data registration may influence feasibility, as both batch-delivery and self-registration are possible.^7^ Self-registration in combination with administrative burden may explain lower feasibility. Our sensitivity analyses of the QI with the lowest feasibility (QI-5; 80%), showed that feasibility is important, particularly for a reliable benchmark. Although imputing missing outcomes may be scientifically appropriate, it remains highly sensitive in hospital comparisons, as providers may interpret it as guesswork that might not present the real world practice and feel unjustly penalized. Therefore, we followed in our study the strategy used by quality registries in the Netherlands, which code missing values to zero, as this provides an incentive for complete registration by individual hospitals. Including the rate of missing values would help provide insight into the overall validity of the data.Nevertheless, we argue that, given the complexity of missing outcome data, the optimal approach to handling missing data should always be determined by experts for each quality registry, or in some cases even for individual QIs.

 Overall, most QIs showed relatively high between-hospital variation, indicating that there is potential for improvement on these indicators. For QIs with low to no between-hospital variation, we have two potential explanations: QIs may have made themselves “unemployable” (ie, healthcare providers adhere so well that a process indicator does not exhibit variation anymore), or there was initially no meaningful variation between hospitals. The first explanation was seen by van Bommel et al^7^ who assessed between-hospital variation in the QI measuring the proportion of patients with breast cancer discussed by the multidisciplinary team, where all hospitals scored >90%. We argue that such an indicator has served its aim and can be de-implemented again. Another example are the QIs irradicality for invasive disease (QI-11) as DCIS (QI-12); both have been collected since 2011, and currently show poor and moderate discriminative ability, respectively.

 It is reassuring that the influence of case-mix on most QIs seems limited, as this influence was high for only four QIs (Pseudo-R^2^≥0.25). The high Pseudo-R^2^ was accompanied by a considerable difference in adjusted and unadjusted scores (high RMSE) for three out of these four (QI-2 – QI-4), emphasising the importance of case-mix adjustment as unadjusted QI scores will be misleading. The influence of case-mix is not surprising here, as these QIs comprise “breast contour preserving treatment” (QI-2), and “immediate breast reconstruction” (QI-3/QI-4), and included case-mix factors like age, BMI and tumour stage are associated with the type of surgery that a patient receives.^20,21^ It is important to emphasise that QIs can have a high pseudo-R^2^ along with little impact of case-mix adjustment on individual hospital scores (low RMSE) if the case-mix variables are evenly distributed across hospitals. Developing specific case-mix adjustment models is essential to avoid unnecessarily complex adjustments. This could also reduce administrative burden by preventing collection of case-mix variables that do not significantly impact hospital comparisons. Nonetheless, case-mix adjusted QI scores should be interpreted cautiously, as there might still be unmeasured confounding: case-mix differences between hospitals that are not measured. Another solution instead of case-mix adjustment could be to stratify patients based on their characteristics^22^; however, this may lead to smaller patient groups and therefore reduce rankability.

 We showed that for nearly all QIs used by the NBCA, rankability was lower than 50% meaning that more than 50% of the observed variation is attributable to chance. Using scores of unreliable QIs for benchmarking may lead to misdirected quality improvement efforts and may potentially have harmful consequences for hospitals. We showed that reporting QI scores over multiple years could be a viable solution to increase the rankability, which is in line with previous work.^23^ Here we encounter an inherent tension between rankability and actionability (meaning that the indicator provides clear guidance for improving performance in daily practice). Reporting data over multiple years contradicts the objective of short cycle feedback, which is inherent in a quality registry, and decreases actionability because feedback information should be as recent as possible to make sure that it contains information about the care currently being delivered. We propose that distinguishing between internal and external (ie, publicly available) QIs is desirable. For external reporting QI scores should span multiple years to increase reliability. Internally, healthcare providers may use the one-year QI scores with detailed information for learning and improving, provided the QI shows moderate rankability. We believe that newly developed indicators should initially be added to the internal QI set before they are publicly reported. Another strategy, currently employed by the NBCA, is to provide QI scores using funnel plots.^7^ Funnel plots show QI scores for individual hospitals incorporating sample size, and show statistically defined control limits.^24,25^ They are easy to use and helpful in correct interpretation of the data, stimulating the conversation towards learning and improvements initiatives.

 There are QIs that still show poor rankability after combining data from three years, especially with those with low number of patients, or with low between-hospital variation. This prompts a discussion on whether information on hospital performance for these QIs should be publicly reported, as this information may be more suitable for internal monitoring over time and as guidance for low-stakes discussions.^4^ Other suggested solutions include excluding QIs with very low or very high event rates,^26^ increase the sample size by clustering hospitals (eg, regions)^27^, and using composite measures,^10,23,28^ although aggregating data by location or indicators may decrease actionability.^29,30^

###  Relation of Quality Indicators to Quality of Care

 Observed differences in QI scores may suggest variations in quality of care, although it is not easy to determine which practice is truly best; high or low frequencies alone do not imply optimal approaches, nor does the average necessarily represent the most effective standard. For some QIs we noted that it is ambiguous how the QI score relates to quality of care. For example, lower irradicality (QI-11/QI-12) does not always imply better quality of care as removing the tumour with large margin may potentially lead to worse patient outcomes in terms of satisfaction and quality of life due to more deformity or increased need for oncoplastic surgery.^31^ The first crucial step before any quantitative assessment is to select relevant QIs and define them unambiguously.

###  Outcome Indicators

 There is a push to increase transparency of outcome indicators, as these are seen as the most valuable information for patients.^32^ Nevertheless, outcomes are generally more influenced by case-mix and less actionable since outcomes do not provide clear guidance for improving. Van Dishoeck et al^33^ showed that outcomes with low event rates in relatively small samples (per hospital) are not suitable for ranking hospitals. This is in line with our results, where three out of four outcome indicators showed moderate to no between-hospital variation and poor rankability (<26%). Additionally, these authors assessed irradicality for invasive disease over one year (2007) and found a rankability of 53%.^33^ Later, Vos et al^5^ calculated rankability for this QI over multiple years (2011-2016) and found a rankability of 22%. Since 2007, changes in the organization of breast cancer care, along with growing awareness of this QI, may have contributed to a decrease in the median irradicality score from 7% in 2007 to 2% in 2023. These changes could explain the 0% rankability observed over one year (2023) and the 10% rankability over three years (2021-2023). This implicates that outcome indicators are not the most suitable candidates for benchmarking. However, it could still be useful for hospitals to monitor their own data and track internal changes over time.

###  Strengths and Limitations

 This is the first data-driven assessment of QIs for breast cancer, providing an objective evaluation of all QIs used in the Netherlands. Our study has some limitations. First, we recoded all missing outcomes to zero in our main analysis. This approach does not affect all QIs homogeneously, and this may have impacted our results. However, as this approach is commonly used in quality registries, to create an incentive for providing data, we have chosen to align as closely as possible with practice. In our sensitivity analyses of QI-5, we performed single imputation of missing outcomes. However, it is questionable if the missingness mechanism is missing at random, as there were hospitals with 100% missingness. Unfortunately we did not have access to extra auxiliary variables for our imputation model which might have influenced the validity of the imputation. Furthermore, we assessed feasibility solely on numerators, which may have led to an underestimation of feasibility. However, since providing data is mandatory for all 71 hospitals delivering breast cancer care in the Netherlands,^7^ we argue that this probably would not have yielded different results. For statistical reasons, rankability could only be calculated for hospitals with more than two patients. Consequently, some hospitals were excluded from certain rankability calculations, which potentially resulted in an overestimation of the rankability. However, as our categorisation was based on values of rankability <50%, 50%-75%, and >75%, we also assume that this limitation did not influence our main results.

###  Implications for Practice and Future Research

 Our results provide a data-driven overview on which QIs are meaningful for assessing the quality of care for breast cancer patients and can inform the Dutch breast cancer registry, an analysis that—to our knowledge—has not been executed before.^1,7^ We recommend using this comprehensive evaluation to refine the QI set for the coming years. Our findings also guide other national registries and international initiatives.^34,35^ The results can provide insights into proposed QIs in such initiatives, for example by identifying reliability concerns related to indicators that focus on subgroups, while considering the national and/or healthcare organization context. Additionally, our practical guide in combination with expert opinion offers valuable support for both national and international benchmarking initiatives.

 If evaluated QI do not meet the criteria, they should serve as fuel for collective learning and improving rather than publicly report them and making providers feel penalized.^4^ Future research should focus on methods to enhance the reliability of breast cancer QIs, such as incorporating composite indicators, and establish the most desirable compromise between reliability and actionability.^23^ Additionally, this assessment approach for evaluation of QIs in hospital settings can be applied to other quality registries using our practical guide (Supplementary file 2), and these criteria should be incorporated when developing new QIs.

## Conclusion

 We demonstrate the importance of thoughtful QI analysis, and therefore these criteria should be considered when developing new QIs. We showed that the QIs employed by the Dutch breast cancer registry fulfilled most criteria, but rankability is a concern that requires specific attention, especially for QIs that are publicly reported. Despite not meeting all criteria, QIs can still offer valuable insights for learning and improvement and could be used as fuel for multidisciplinary conversation. Furthermore, we provide a practical guide for performance analysis of QIs.

## Acknowledgements

 The authors thank all, registrar and healthcare professionals for data registration and development of the NBCA.

## Ethical issues

 The board and scientific committee of the NBCA reviewed and approved the data request for this study. According to Dutch legislation, informed consent from individual patients was not required for this study.

## Conflicts of interest

 Authors declare that they have no conflicts of interest.

## Data sharing statement

 We used data from the NABON breast cancer audit (NBCA), a nationwide clinical audit. Data are available upon reasonable request.

## Collaborators the NBCA Consortium

 The NBCA Consortium consists of the following participants, displayed with their affiliations:

**Alwine A. Hellingman** (Department of Surgery, Tergooi Medical Centre, Hilversum, the Netherlands), **Anne Brecht Francken** (Department of Surgery, Isala klinieken, Zwolle, the Netherlands), **Annemiek Doeksen** (Department of Surgery, Antonius Ziekenhuis Nieuwegein, Nieuwegein, the Netherlands), **Carolien H.M. van Deurzen** (Department of Pathology, Erasmus MC Cancer Institute, Erasmus University Medical Centre, Rotterdam, the Netherlands), **Cristina Guerrero Paez** (Dutch Breast Cancer Society (BVN), Utrecht, the Netherlands), **Danielle M. de Leeuw **(Department of Surgery, Ziekenhuisgroep Twente, Almelo/Hengelo, the Netherlands), **Djaëlla V. Hooiveld** (Department of Surgery, Zaans Medical Centre, Zaandam, the Netherlands), **Ernst. J.P. Schoenmaeckers** (Department of Surgery, Meander Medical Centre, Amersfoort, the Netherlands), **Els Van Dessel** (Department of Surgery, ZorgSaam hospital, Terneuzen, the Netherlands), **Emily L. Postma** (Department of Surgery, St. Antonius Hospital, Nieuwegein, the Netherlands), **Esther D. van den Ende** (Department of Surgery, Saxenburgh Medical Centre, Hardenberg, the Netherlands), **Gabor S.A. Abis** (Department of Surgery, Meander Medical Centre, Amersfoort, the Netherlands), **Grietje Bouma** (Department of Internal Medicine, Nij Smellinghe Hospital, Drachten, the Netherlands), **Henriette A. Schuttevaer** (Department of Surgery, Martini Hospital, Groningen, the Netherlands), **Hinne A. Rakhorst** (1. Department of Plastic, Reconstructive, and Hand Surgery, Medisch Spectrum Twente, Enschede, The Netherlands. 2. Department of Plastic, Reconstructive, and Hand Surgery, Ziekenhuisgroep Twente, Almelo, The Netherlands),** Ilse Jannink** (Department of Surgery, Hagaziekenhuis, The Hague, the Netherlands), **Ingrid Kappers **(Department of Surgery, Tjongerschans ziekenhuis, Heerenveen, the Netherlands), **Janneke Verloop** (Department of Research and Development, Netherlands Comprehensive Cancer Organisation (IKNL), Utrecht, the Netherlands), **Joost Nonner** (Breast Cancer Center South Holland South, Ikazia Hospital, Rotterdam), **Klaartje van Engelen** (Department of Human Genetics, Amsterdam UMC and University of Amsterdam, Amsterdam), **Linda de Munck** (Department of Research and Development, Netherlands Comprehensive Cancer Organisation (IKNL), Utrecht, the Netherlands), **Loes F.S. Kooreman** (Department of Pathology, Maastricht University Medical Center, GROW – School for Oncology and Reproduction, Maastricht University, Maastricht, The Netherlands), **Marianne R.F. Bosscher** (Department of Surgery, Ommelander Hospital Groningen, Scheemda, the Netherlands), **Marian B.E. Menke-Pluijmers** (Department of Surgery, Albert Schweitzer Hospital, Dordrecht, Netherlands), **Margrethe schlooz-vries (**Department of Surgery, Radboud University Medical Centre, Nijmegen, the Netherlands), **Miriam L. Hoven-Gondrie** (Department of Surgery, Gelderse Vallei, Ede, the Netherlands), **Martinus A. Beek** (Department of Surgery, Isala klinieken, Zwolle, the Netherlands), **Marije J. Hoornweg **(Department of Plastic Surgery, Netherlands Cancer Institute - Antoni van Leeuwenhoek Hospital, Amsterdam, the Netherlands), **Menno H. Raber **(Department of Surgery, Saxenburgh Medical Centre, Hardenberg, the Netherlands), **Milou H. Martens** (Department of Surgery, Laurentius Hospital, Roermond, the Netherlands), **Barbara G. Molenkamp** (Department of Surgery, Diakonessenhuis, Utrecht, the Netherlands), **Patricia Jansen** (Department of Surgery, Elizabeth Tweesteden Hospital, Tilburg, the Netherlands), **Ramon R.J.P. van Eekeren** (Department of Surgery, Rijnstate Hospital, Arnhem, the Netherlands), **Thomas Schok** (Department of Surgery, VieCuri Medical Centre, Venray, the Netherlands), **Robert-Jan Schipper **(Department of Surgery, Catharina Hospital, Eindhoven, the Netherlands), **Titia E. Lans** (Department of Surgery, Admiraal de Ruyterziekenhuis, Goes and Vlissingen, the Netherlands), **Vivianne C.G. Tjan-Heijnen **(Department of Medical Oncology, GROW, Maastricht University Medical Centre, Maastricht, the Netherlands), **Yvonne L. J. Vissers **(Department of Surgery, Zuyderland Medical Centre, Heerlen, the Netherlands).

## Supplementary files


Supplementary file 1 contains Tables S1-S3 and Figures S1-S2.

Supplementary file 2. Practical Guide for Comprehensive Quality Indicator Evaluation (Including R Code).

Supplementary file 3. Sensitivity analysis. This supplementary file contains Figure S3 and Table S4.
