# Supplementary material for: Comprehensive Evaluation of Quality Indicators: Analyzing the Dutch Breast Cancer Audit
Source: Int J Health Policy Manag. 2025 Oct 1;14:8943. doi: 10.34172/ijhpm.8943 (PMC12958241; doi:10.34172/ijhpm.8943)
Supplement: Supplementary file 1 — contains Tables S1-S3 and Figures S1-S2. [file ijhpm-14-8943-s001.pdf]

Article title: Comprehensive Evaluation of Quality Indicators: Analyzing the Dutch Breast Cancer Audit

Journal name: International Journal of Health Policy and Management (IJHPM)

Authors' information: Elfi M. Verheul[1,2]¶*, Margrietha van der Linde[1]¶, Hester F. Lingsma[1], Elvira Vos[3], Sabine Siesling[4,5], Linetta B. Koppert[6], NBCA Consortium[#]

[1]Center for Medical Decision Making, Department of Public Health, Erasmus University Medical Center, Rotterdam, The Netherlands.

[2]Dutch Institute for Clinical Auditing, Leiden, The Netherlands.

[3]Department of Surgery, Rhode Island Hospital, Warren Alpert Medical School of Brown University, Providence, RI, USA.

[4]Department of Research, Netherlands Comprehensive Cancer Organization (IKNL), Utrecht, The Netherlands.

[5]Department of Health Technology and Services Research, Technical Medical Centre, University of Twente, Enschede, The Netherlands.

[6]Department of Surgery, Erasmus MC Cancer Institute, Rotterdam, The Netherlands.

***Correspondence to:** Elfi M. Verheul; Email: e.verheul@erasmusmc.nl

Citation: Verheul EM, van der Linde M, Lingsma HF, et al. Comprehensive evaluation of quality indicators: analyzing the Dutch breast cancer audit. Int J Health Policy Manag. 2025;14:8943. doi:10.34172/ijhpm.8943

#A full list of the collaborators of the NBCA Consortium is provided at the end of the article.

¶ Both authors contributed equally to this paper.

Supplementary file 1

**Figure S1**	Criteria for quality indicators as summarized by The European Observatory on Health Systems and Policies
**Table S1**	Overview of models for calculating the rankability of the quality indicators
**Table S2**	Influence of case-mix adjustment on hospital comparisons for breast cancer patients treated surgically in the Netherlands between 2021-2023.
**Table S3**	Rankability of quality indicators calculated over 1 year vs calculated over 3 years.
**Figure S2**	Unadjusted and Adjusted Observed-Expected ratios for each Quality indicator

Box 3.1 *Criteria for indicators*

Relevance

- *Impact of disease or risk on health and health expenditures.* What is the impact on health and on health expenditure associated with each disease, risk or patient group?
- *Importance.* Are relevant stakeholders concerned about the quality problem and have they endorsed the indicator?
- *Potential for improvement.* Does evidence exist that there is less-than-optimal performance, for example, variation across providers?
- *Clarity of purpose and context.* Are the purpose of the indicator and the organizational and healthcare contexts clearly described?

Scientific soundness

- *Validity.* Does the indicator measure what it is intended to measure? The indicator should make sense logically and clinically (face validity); it should correlate well with other indicators of the same aspects of the quality of care (construct validity) and should capture meaningful (i.e. evidence-based) aspects of the quality of care (content validity).
- *Sensitivity and specificity.* Does the indicator detect only a few false positives and false negatives?
- *Reliability.* Does the measure provide stable results across various populations and circumstances?
- *Explicitness of the evidence base.* Is scientific evidence available to support the measure (for example, systematic reviews, guidelines, etc.)?
- *Adequacy of the appraisal concept.* Are reference values fit for purpose, and do they allow identification of good and bad providers?

Feasibility

- *Previous experience.* Is the measure in use in pilot programmes or in other countries?
- *Availability of required data across the system.* Can information needed for the measure be collected in the scale and timeframe required?
- *Cost or burden of measurement.* How much will it cost to collect the data needed for the measure?
- *Capacity of data and measure to support subgroup analyses.* Can the measure be used to compare different groups of the population (for example, by socioeconomic status to assess disparities)?

Meaningfulness

- *Comparability:* does the indicator permit meaningful comparisons across providers, regions, and/or countries?
- *User-friendliness:* is the indicator easily understood and does it relate to things that are important for the target audience?
- *Discriminatory power:* does the indicator distinguish clearly between good and bad performers?

Sources: Hurtado, Swift & Corrigan, 2001; Mainz, 2003; Kelley & Hurst, 2006; de Koning, Burgers & Klazinga, 2007; Evans et al., 2009; Lüngen & Rath, 2011; IQTIG, 2018; NQF, 2019b

Figure S1 Criteria for quality indicators as summarized by The European Observatory on Health Systems and Policies (14).

Table S1: Overview of models for calculating the rankability of the quality indicators, including details on how many hospitals were excluded and why and which case-mix variables were excluded in the calculation of rankability, when this was necessary for model convergence.

Quality indicator	N hospitals	Reason(s) excluding hospitals	Excluded predictors in case-mix adjustment model
**QI-2**			
QI-2A	71	-	
QI-2B	71	-	
QI-2C	71	-	
QI-2D	71	-	
**QI-3A**			
QI-3A	71	-	
QI-3B	71	-	
QI-3C	38	Excluded hospitals that do not perform autologous reconstruction (N=33)	
QI-3D	12	Excluded hospitals that do not performed the combination of prosthesis and autologous reconstruction (N = 59). Questionable if this is fair (rankability is 0, but as 0% officially also counts we can consider to do NA	
QI-3E	-	-	
**QI-4**			
QI-4A	65	Excluded hospitals that did treat less than 2 patients in 2023 (N=6)	N-stage & T-stage (because DCIS)
QI-4B	65	Excluded hospitals that did treat less than 2 patients in 2023 (N=6)	N-stage & T-stage (because DCIS)
QI-4C	-	Groups are too small	
QI-4D	-	Groups are too small	
QI-4	-	Groups are too small	
**QI-5**	71		
**QI-6**	69	Excluded hospitals that did treat less than 5 patients in 2023 (N=2)	
**QI-7**			
QI-7A	71		
QI-7B	71		
QI-7C	71		
QI-7D	69	Excluded hospitals with 0 patients in the denominator (N=2)	
**QI-9**	71	-	
**QI-10**	66	Excluded hospitals that did treat less than 2 patients in 2023 (N=5)	
QI-11	69	Excluded hospitals that did treat less than 2 patients in 2023 (N=2)	
QI-12	70	Excluded hospitals that did treat less than 2 patients in 2023 (N=1)	Age, N-stage, T-stage (because DCIS)
**QI-13**			
QI-13A	71		
QI-13B	66	Excluded hospitals with 0 patients in the denominator(N=5)	
QI-13C	71		
**QI-14**	67	Excluded hospitals with 0 patients in a clinical study (N=4)	
**QI-15**			
QI-15A	71		
QI-15B	71		
QI-15C	71		
**QI-16**			
QI-16A	71		
QI-16B	71		
QI-16C	69	Excluded hospitals that did treat less than 2 patients in 2023 (N=2)	
QI-17	65	Excluded hospitals with less than 2 patients (N=6)	
QI-18	51	Excluded hospitals with less than 1 patient (N=20)	N-stage, T-stage (because DCIS)
**QI-19**			
QI-19A	71		
QI-19B	65	Excluded hospitals that did treat less than 2 patients in 2023 (N=6)	
QI-19C	61	Excluded hospitals that did treat less than 2 patients in 2023 (N=10)	

Table S2: Influence of case-mix adjustment on hospital comparisons for breast cancer patients treated surgically in the Netherlands between 2021-2023.

Quality indicator[1]	(Pseudo) R-squared[2]	RMSE[3]
**QI-2**		
QI-2A	0.26	0.021
QI-2B	0.53	0.081
QI-2C	0.35	0.122
QI-2D	0.28	0.384
**QI-3A**		
QI-3A	0.38	0.193
QI-3B	0.33	0.209
QI-3C	0.10	0.412
QI-3D	0.05	0.273
QI-3E	0.09	0.252
**QI-4**		
QI-4A	0.36	0.240
QI-4B	0.27	0.221
QI-4C	0.07	0.595
QI-4D	0.05	0.283
QI-4	0.10	0.578
**QI-5**	0.02	0.035
**QI-6**	0.10	0.010
**QI-7**		
QI-7A	0.03	0.009
QI-7B	0.03	0.012
QI-7C	0.04	0.017
QI-7D	0.02	0.013
**QI-9**	0.07	0.014
**QI-10**	0.12	0.038
QI-11	0.06	0.126
QI-12	0.03	0.076
**QI-13**		
QI-13A	0.02	0.007
QI-13B	0.02	0.025
QI-13C	0.02	0.008
**QI-14**	0.04	0.121
**QI-15**		
QI-15A	0.01	0.045
QI-15B	0.01	0.036
QI-15C	0.02	0.077
**QI-16**		
QI-16A	0.03	0.043
QI-16B	0.03	0.054
QI-16C	0.03	0.049
QI-17	0.10	0.204
QI-18	0.05	0.099
**QI-19**		
QI-19A	0.17	0.037
QI-19B	0.14	0.038
QI-19C	0.25	0.073

QI = quality indicator. [1]See Table 1 for exact definitions of all quality indicators. [2]For continuous outcomes QI7 and QI12 the R-squared is presented instead of the Pseudo R-squared. [3]The root mean squared error (RMSE) measures the impact of case-mix adjustment on between-hospital differences in quality indicator scores and is defined as the square root of the average squared deviance per hospital (see methods). No effect of case-mix correction corresponds to an RMSE of 0.

Table S3: Rankability of quality indicators calculated over 1 year vs calculated over 3 years.

Quality indicator[1]	N patients[2]	Rankability %	N patients[2]	Rankability %
**Year(s) of data**	**2023**	**2023**	**2021-2023**	**2021-2023**
**QI-2**				
QI-2A	13502	32	43012	68
QI-2B	13502	22	43012	55
QI-2C	13502	16	43012	59
QI-2D	13502	31	43012	75
**QI-3**				
QI-3A	3881	37	13015	75
QI-3B	3881	33	13015	74
QI-3C	3881	32	13015	65
QI-3D	3881	16	13015	34
QI-3E	3881	NA	13015	NA
**QI-4**				
QI-4A	533	8	1627	17
QI-4B	533	8	1627	29
QI-4C	533	NA	1627	NA
QI-4D	533	NA	1627	NA
QI-4E	533	NA	1627	NA
**QI-5**	1812	61	7976	83
**QI-6**	1466	0	4574	16
**QI-7**				
QI-7A	13976	66	42378	86
QI-7B	3792	39	11786	66
QI-7C	9532	66	28461	87
QI-7D	652	27	2131	55
**QI-8**	15596	-		-
**QI-9**	2773	69	10479	74
**QI-10**	535	0	1915	14
**QI-11**	7528	0	22461	10
**QI-12**	1384	15	4032	0
**QI-13**				
QI-13A	10533	52	34652	85
QI-13B	575	23	2058	53
QI-13C	9412	56	31418	85
**QI-14**	15596	30	49359	53
**QI-15**				
QI-15A	15596	0	49359	23
QI-15B	11187	13	34839	42
QI-15C	4372	0	14341	0
**QI-16**				
QI-16A	3785	24	14152	55
QI-16B	2773	25	10479	48
QI-16C	1406	0	5414	31
**QI-17**	9516	67	29769	70
**QI-18**	1347	37	3942	48
**QI-19**				
QI-19A	1408	0	4300	39
QI-19B	889	53	2677	62
QI-19C	519	20	1623	42

QI = quality indicator. [1]See Table 1 for exact definitions of all quality indicators. [2]The number of patients in denominator. The colours indicate poor (orange), moderate (yellow) and good (green) performance on the selected criteria.

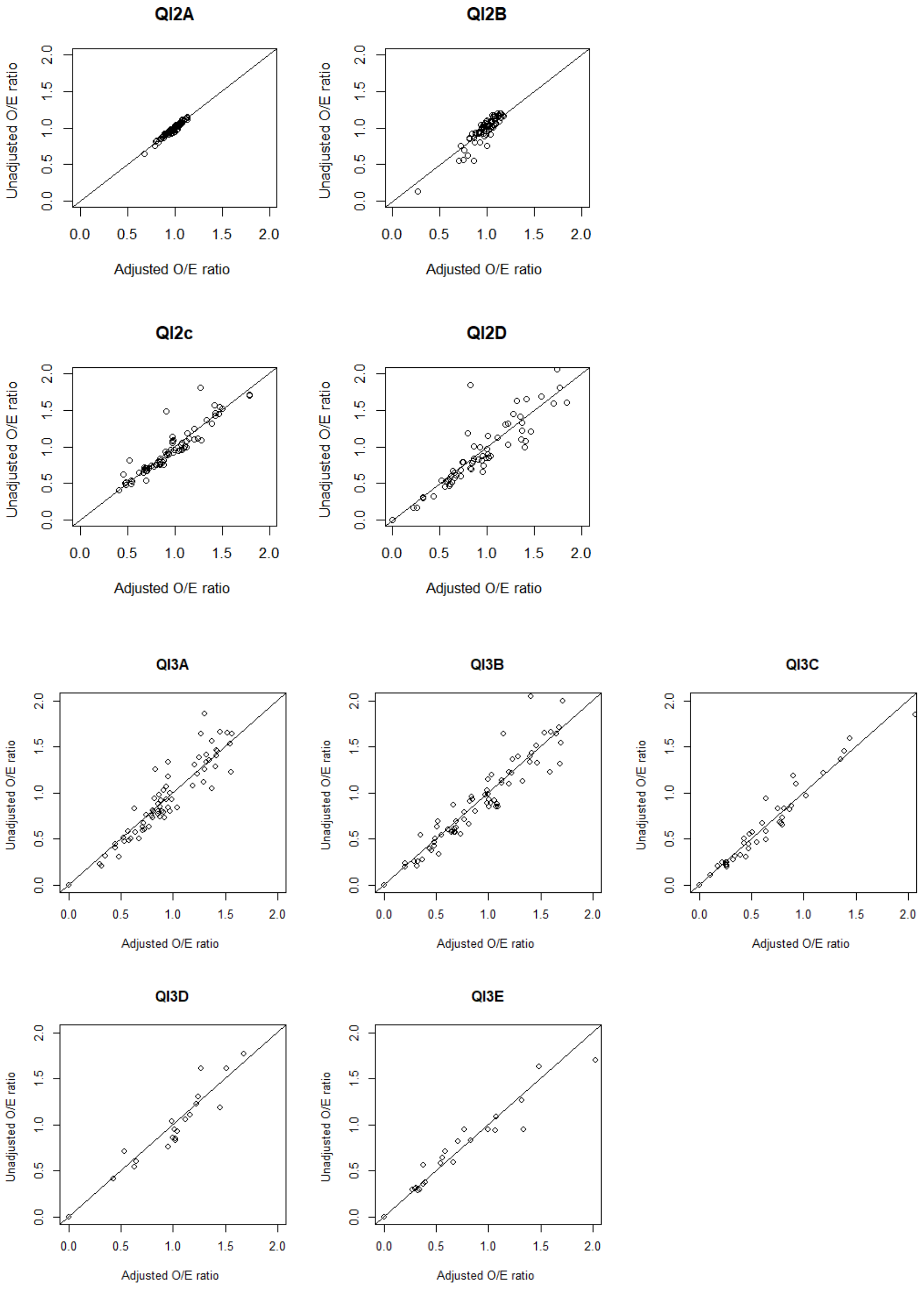

Figure S2*: Unadjusted and Adjusted Observed-Expected ratios for each Quality indicator (QI). Each dot represents a hospital. The X-axis shows the O/E ratio before case-mix adjustment, while the Y-axis shows the O/E ratio after case-mix adjustment. Deviation from the diagonal presents the effect of case-mix adjustment.*

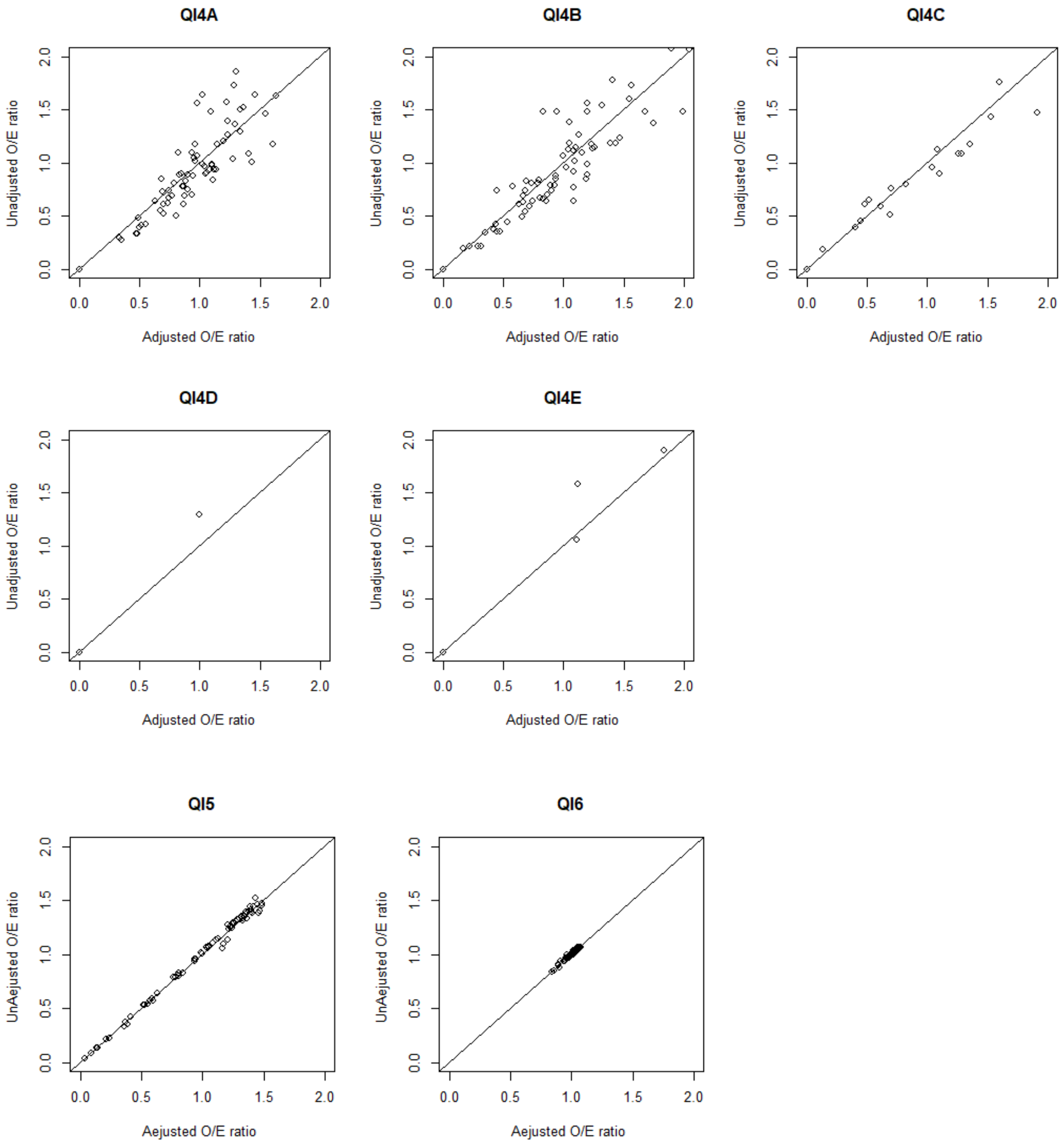

***Figure S2**: Unadjusted and Adjusted Observed-Expected ratios for each Quality indicator (QI). Each dot represents a hospital. The X-axis shows the O/E ratio before case-mix adjustment, while the Y-axis shows the O/E ratio after case-mix adjustment. Deviation from the diagonal presents the effect of case-mix adjustment.*

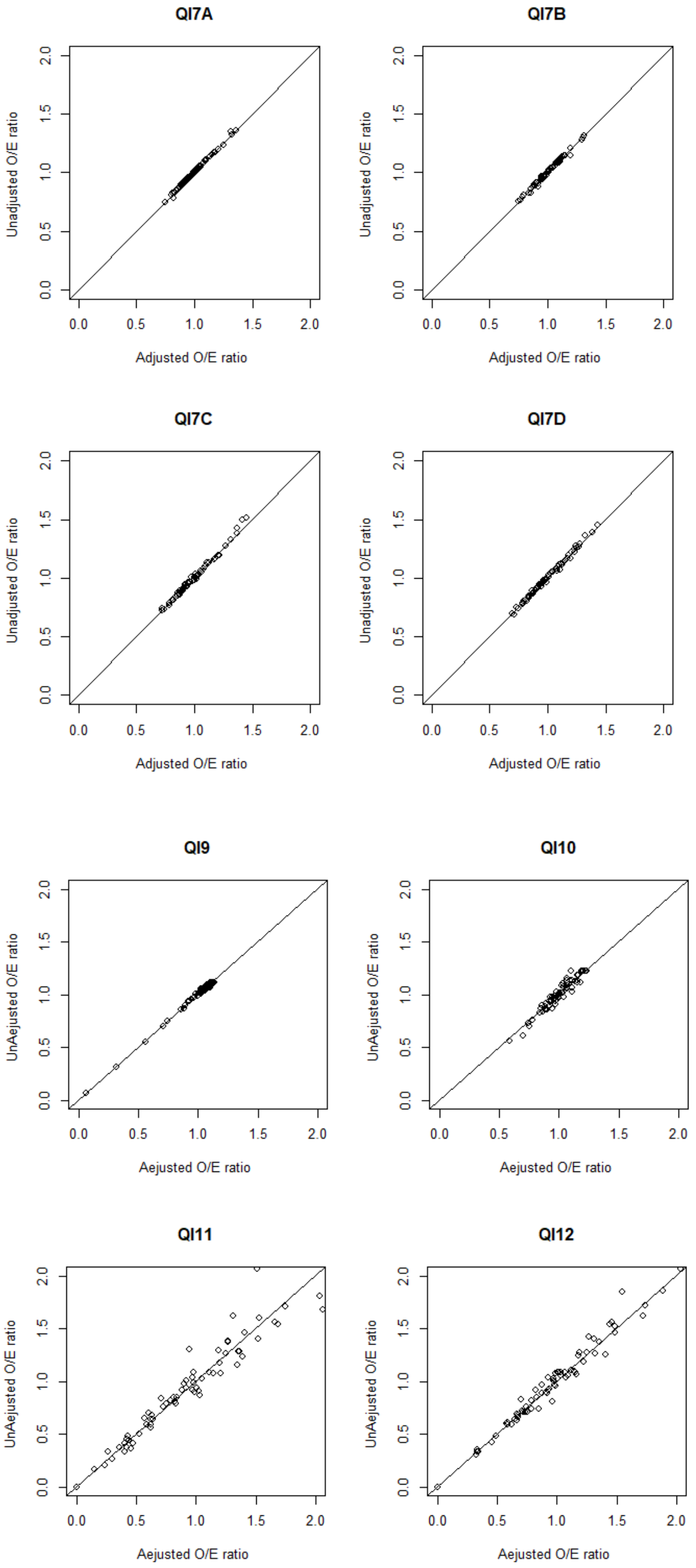

***Figure S2**: Unadjusted and Adjusted Observed-Expected ratios for each Quality indicator (QI). Each dot represents a hospital. The X-axis shows the O/E ratio before case-mix adjustment, while the Y-axis shows the O/E ratio after case-mix adjustment. Deviation from the diagonal presents the effect of case-mix adjustment.*

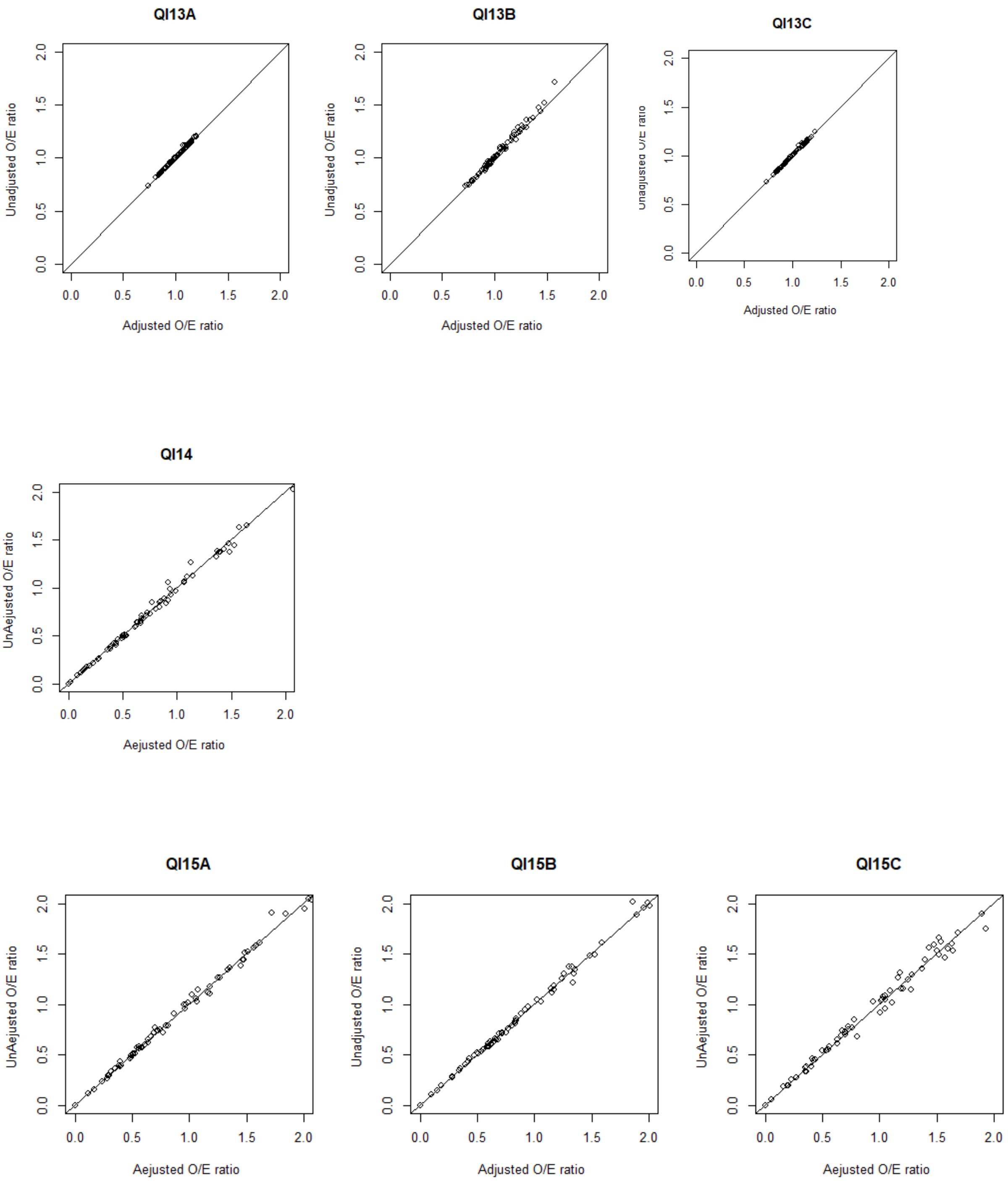

***Figure S2**: Unadjusted and Adjusted Observed-Expected ratios for each Quality indicator (QI). Each dot represents a hospital. The X-axis shows the O/E ratio before case-mix adjustment, while the Y-axis shows the O/E ratio after case-mix adjustment. Deviation from the diagonal presents the effect of case-mix adjustment.*

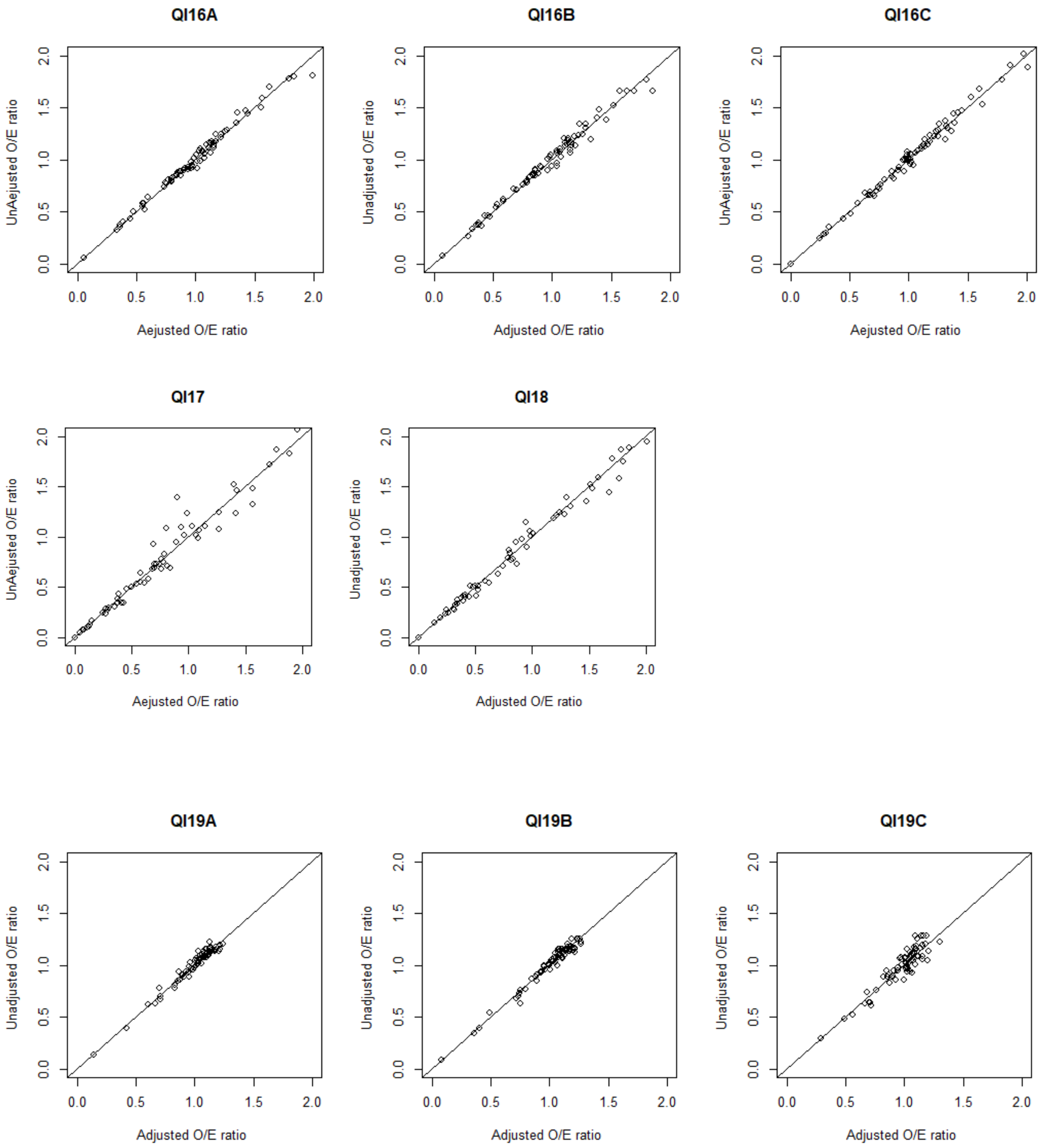

***Figure S2**: Unadjusted and Adjusted Observed-Expected ratios for each Quality indicator (QI). Each dot represents a hospital. The X-axis shows the O/E ratio before case-mix adjustment, while the Y-axis shows the O/E ratio after case-mix adjustment. Deviation from the diagonal presents the effect of case-mix adjustment.*
