# Supplementary material for: Comprehensive Evaluation of Quality Indicators: Analyzing the Dutch Breast Cancer Audit
Source: Int J Health Policy Manag. 2025 Oct 1;14:8943. doi: 10.34172/ijhpm.8943 (PMC12958241; doi:10.34172/ijhpm.8943)
Supplement: Supplementary file 2 — Practical Guide for Comprehensive Quality Indicator Evaluation (Including R Code). [file ijhpm-14-8943-s002.pdf]

Article title: Comprehensive Evaluation of Quality Indicators: Analyzing the Dutch Breast Cancer Audit

Journal name: International Journal of Health Policy and Management (IJHPM)

Authors' information: Elfi M. Verheul[1,2]¶*, Margrietha van der Linde[1]¶, Hester F. Lingsma[1], Elvira Vos[3], Sabine Siesling[4,5], Linetta B. Koppert[6], NBCA Consortium[#]

[1]Center for Medical Decision Making, Department of Public Health, Erasmus University Medical Center, Rotterdam, The Netherlands.

[2]Dutch Institute for Clinical Auditing, Leiden, The Netherlands.

[3]Department of Surgery, Rhode Island Hospital, Warren Alpert Medical School of Brown University, Providence, RI, USA.

[4]Department of Research, Netherlands Comprehensive Cancer Organization (IKNL), Utrecht, The Netherlands.

[5]Department of Health Technology and Services Research, Technical Medical Centre, University of Twente, Enschede, The Netherlands.

[6]Department of Surgery, Erasmus MC Cancer Institute, Rotterdam, The Netherlands.

***Correspondence to:** Elfi M. Verheul; Email: e.verheul@erasmusmc.nl

Citation: Verheul EM, van der Linde M, Lingsma HF, et al. Comprehensive evaluation of quality indicators: analyzing the Dutch breast cancer audit. Int J Health Policy Manag. 2025;14:8943. doi:10.34172/ijhpm.8943

#A full list of the collaborators of the NBCA Consortium is provided at the end of the article.

¶ Both authors contributed equally to this paper.

Supplementary file 2. Practical Guide for Comprehensive Quality Indicator Evaluation (Including R Code)

This practical guide will walk you through conducting a comprehensive evaluation of the quality indicators (QIs) in your registry, using the criteria feasibility, discriminative ability, validity, and reliability. Keep in mind that qualitative criteria are not included in this guide, but factors such as indicator importance, clear definitions, and accurate registration are essential for unambiguous interpretation before conducting qualitative evaluation.

Before proceeding with the step-by-step instructions, ensure the following:

1. Data cleaning: This involves detecting, correcting, or removing errors and inconsistencies in your data to improve its quality. Key tasks include removing duplicate rows, correcting outliers, and standardizing the data.
2. Definition of numerators and denominators for QIs: Clearly define the numerators (the number of cases meeting the criteria) and denominators (the total number of cases) for all the quality indicators you plan to assess. Add whether each numerator and denominator is `TRUE` or `FALSE` per patient (use the variables and/or methods that are needed for calculating the numerator and denominator to align with practice).
   Example:
   ```
   df$denominator_QI_1 <- df$variable_A %in% c(1, 2, 3) & df$variable_B %in% c(1, 2)
   df$numerator_QI_1 <- df$variable_C == 1 & df$denominator_QI_1 == TRUE
   ```
3. Missing values: In this step you will consider your approach to handling missing values. Regardless of the approach, it is important to also maintain a version of the cleaned dataset with definitions, preserving the original missing values.
   - Missing baseline characteristics: Determine which method you will use, options include methods such as complete case analysis, single or multiple imputation. Take into account the different mechanisms of missing data: missing completely at random (MCAR), missing at random (MAR) and missing not at random (MNAR).
   - Missing values in outcomes: Consider how to handle missing values in the outcomes and explore possible solutions. This might include following the method of the clinical registry, excluding patients with missing values, using imputation methods or conducting sensitivity analyses as recent research shows that complete case analysis can bias results. [1]
4. Consideration of analysis period: It is important to align the analysis period with the practical reporting periods of the QIs to adhere as closely as possible to real-world practices. For example, in the Dutch breast cancer registry, QI scores are (publicly) reported over a one-year period, so we selected one year for our analysis period. However, to assess the influence of case-mix, we used data spanning multiple years to achieve more robust results.

On page 10 you can find an empty table to fill in all your results.

1. van Linschoten RCA, Amini M, van Leeuwen N, et al. Handling missing values in the analysis of between-hospital differences in ordinal and dichotomous outcomes: a simulation study. BMJ Qual Saf 2023;32(12):742-49. doi: bmjqs-2023-016387 [pii] 10.1136/bmjqs-2023-016387 [published Online First: 2023/09/22]

Step-by-step guide

Feasibility

The performance of quality indicators largely depends on the quality and availability of data. The feasibility criterion evaluates the availability of data in the numerator, which is the percentage of data that is not missing. Note that this approach may slightly underestimate the true feasibility, as there could also be missing values in the denominator. However, it is impossible to determine which patients should be included in the denominator when the necessary values are missing.

$$Quality\ indicator\ = \frac{Numerator}{Demoninator}$$

In the step-by-step guide below the overall feasibility is calculated. If you want to calculate the range of feasibility per hospital, you can use the `group_by()` function in R.

Step 1: Install and load packages that are needed.

```
install.packages("readr")
install.packages("dplyr")
library(readr)
library(dplyr)
```

Step 2: Load your dataset into R. Choose the correct way to load your data, depending on file type. As we're calculating the percentage of the data that is not missing, it is important that you load the cleaned dataset in which you didn't exclude or impute patients with missing outcomes. We assume that this data contains the right analysis period and all the numerator and denominator variables.

```
df <- read.csv("your_dataset_with_missing_values.csv")
```

Step 3: Select patients of each denominator for each hospital and create separate data frame (example provided with one QI).

```
rows_denominator_QI_1 <- df %>%
  filter(denominator_QI_1 == TRUE)
```

Step 4: Determine number of rows in dataframe

```
Rows_denominator_QI_1 <- nrow(rows_denominator_QI_1)
```

Step 5: Calculate number of missings in numerator. In this example below, the numerator is determined based on three variables: variable_A, variable_B and variable_C.

```
Numerator_QI_1_missing <- rows_denominator_QI_1 %>% filter(
      (is.na(variable_A) | is.na(variable_B) | is.na(variable_C))
```

Step 6: Calculate number of rows that is not missing

```
Rows_numerator_QI_1 <- Rows_denominator_QI_1 – nrow(Numerator_QI_1_missing)
```

Step 7: Calculate percentage of data available (feasibility).

```
Feasibility_QI_1 <- (Rows_numerator_QI_1 / Rows_denominator_QI_1) * 100
Feasibility_QI_1
```

Step 8: Classify data completeness. Feasibility of the numerator above 90% is classified as good, between 70-90% as moderate, and below 70% as poor.

Step 9: Fill in the table at the end of this practical guide. If feasibility <25%, you can decide to exclude the QI for further evaluation on remaining criteria.

Discriminative ability

Discriminative ability encompasses the ability of a QI to distinguish in hospital performance. If there is only little between-hospital variation, there may be small improvement possibilities, while wide hospital variation offers more room for improvement. Discriminative ability is quantified by the extent of between-hospital variation, which can be reported with median and interquartile range (IQR).

Discriminative ability is calculated differently for binary and continuous indicators.

Binary indicators:

This is an example of QI 1, consisting of QI 1A and QI 1B with the same denominator. This analysis can be repeated for the remaining binary QIs.

Step 1: Install and load packages that are needed.

```
install.packages("readr ")
install.packages("dplyr ")
install.packages("knitr ")
library(readr)
library(dplyr)
library(knitr)
```

Step 2: Load your dataset into R. We assume that this data contains the right analysis period and all the numerator and denominator variables. Choose the correct way to load your data, depending on file type.

```
df <- read.csv("your_dataset_with_missing_values.csv")
```

Step 3: Define function to calculate summary of between-hospital variation. This function will calculate median, Q1, Q3, IQR, minimum, maximum and range.

```
calculate_summary_outcome <- function(data, variable_name) {
  numeric_data <- as.numeric(data[[variable_name]])
  median_var <- round(median(numeric_data, na.rm = TRUE), 2)
  q1_var <- round(quantile(numeric_data, 0.25, na.rm = TRUE), 2)
  q3_var <- round(quantile(numeric_data, 0.75, na.rm = TRUE), 2)
  iqr_var <- round(q3_var - q1_var, 2)
  range_var <- round(max(numeric_data, na.rm = TRUE) - min(numeric_data,
na.rm = TRUE), 2)
  min_var <- round(min(numeric_data, na.rm = TRUE), 2)
  max_var <- round(max(numeric_data, na.rm = TRUE), 2)

  return(data.frame(
    Variable = variable_name,
    Median = median_var,
    Q1 = q1_var,
    Q3 = q3_var,
    IQR = iqr_var,
    Range = range_var,
    Minimum = min_var,
    Maximum = max_var
  ))
}
```

Step 4: Select patients of each denominator and create separate data frame.

```
QI_1 <- subset(df, denominator_QI_1 == TRUE)
```

Step 5: Use the `calculate_summary` function to calculate between-hospital variation for each QI separately in the dataframe that you created in step 4. In this part of the analysis, the hospital identifier is

used to distinguish between hospitals using the `group_by()` function. First, a QI score will be calculated for each hospital. From these QI scores, the median, IQR, and range will be established.

```
summary_table_numerator_1A <- QI_1  %>%
  group_by(hospital_id) %>%
  summarise(numerator_1A = sum(numerator_1A == TRUE, na.rm = TRUE) / n() *
100) %>%
  { calculate_summary_outcome(., "numerator_1A") }

summary_table_numerator_1B <- QI_1  %>%
  group_by(hospital_id) %>%
  summarise(numerator_1B = sum(numerator_1B == TRUE, na.rm = TRUE) / n() *
100) %>%
  { calculate_summary_outcome(., "numerator_1B") }
```

Step 6: Create table for both QI1A and QI1B, showing between-hospital variation median, IQR and range (min-max).

```
QI1 <-rbind(summary_table_numerator_1A, summary_table_numerator_1B)

kable(QI1, format = "markdown", row.names = FALSE)
```

Step 7: Classify between-hospital variation as poor if IQR<5, as moderate if IQR is between 5-10, and as good if IQR>10.

Step 8: Fill in the table at the end of this practical guide.

Continuous indicators:

This is an example of QI 2, a continuous indicator. In this example, the numerator is defined as the number of days and the denominator as `TRUE` or `FALSE`. This analysis can be repeated for the remaining continuous QIs.

Step 1: Install and load packages that are needed.

```
install.packages("readr ")
install.packages("dplyr ")
install.packages("knitr ")
library(readr)
library(dplyr)
library(knitr)
```

Step 2: Load your dataset into R. We assume that this data contains the right analysis period and all the numerator and denominator variables. Choose the correct way to load your data, depending on file type.

```
df <- read.csv("your_dataset_with_missing_values.csv")
```

Step 3: Select patients of each denominator and create separate data frame.

```
QI_2 <- subset(df, denominator_QI_2 == TRUE)
```

Step 4: Calculate summary statistics (median, Q1, Q3, IQR, minimum, maximum and range) for QI 2.

```
summary_table_QI2 <- QI_2 %>%
  summarise(Variable = "numerator_2",
            Median = median(teller12a, na.rm = TRUE),
            Q1 = quantile(teller12a, 0.25, na.rm = TRUE),
            Q3 = quantile(teller12a, 0.75, na.rm = TRUE),
```

```
IQR = IQR(teller12a, na.rm = TRUE),
 Range  =  max(teller12a,  na.rm  =  TRUE)  -  min(teller12a,
 na.rm = TRUE),
Minimum = min(teller12a, na.rm = TRUE),
Maximum = max(teller12a, na.rm = TRUE))
```

Step 5: Classify between-hospital variation as poor if IQR<5, as moderate if IQR is between 5-10, and as good if IQR>10. Depending on the type and unit of the indicator, you may choose to express this in standard deviations.

Step 6: Fill in the table at the end of this practical guide.

Validity

For this criterion, we assess the impact of adjustment of baseline patient and disease characteristics (i.e., case-mix). For this part, you can use data from multiple years to yield more robust results. The influence of case-mix is quantified using the Nagelkerke's pseudo-R^2 for the binary QIs and the R^2 for the continuous QIs. In the context of between-hospital comparisons little impact of case-mix adjustment is favourable, as with high case-mix influence observed between-hospital variation in the QI score is more likely due to differences in underlying patient population rather than quality of care provided.

When patient populations are comparable across hospitals, the influence of adjustment on hospital comparisons is likely to be minimal. Therefore, you can calculate the root mean squared error (RMSE), which quantifies the total deviation from the diagonal if adjusted and unadjusted O/E ratios are plotted in a scatterplot (see methods in main article for in depth explanation). A lower RMSE indicates a smaller effect of case-mix adjustment on hospital comparisons. If the hospitals are exactly on the diagonal, there is no effect of case-mix adjustment (RMSE = 0).

The step-by-step guide proved code for example QI with variable name "QI_1".

Step 1: Consider through either/both expert opinion and literature which variables available in your data could be potential case-mix factors.

Step 2: Install and load packages that are needed.

```
install.packages("readr ")
install.packages("dplyr ")
install.packages("mice ")
install.packages("rms ")
library(readr)
library(dplyr)
library(mice)
library(rms)
```

Step 3: Load your dataset into R. We assume that this is data in which missing baseline characteristics are imputed and that the data contains the right analysis period and all the numerator and denominator variables. Choose the correct way to load your data, depending on file type.

```
data <- read_rds("your_dataset.rds")
```

Step 4: Select patients of each denominator and create separate data frame

```
QI_1 <- subset(data, denominator_QI_1 == TRUE)
```

Step 5: Fit a regression model. Use logistic regression for binary QIs (in example below QI1), linear regression for continuous QIs (in example below QI2).

```
#logistic regression
qi_1 <-lrm(numerator1 ~  case-mix_variables, data= QI_1, x=TRUE, y=TRUE)

#check pseudo R2
qi_1

#if continuous QI, then apply linear regression
qi_2 <- lm(numerator2 ~  case-mix_variables, data=QI_2)

#check R2
qi_2
```

Step 6: Classify the impact of case-mix adjustment: A (pseudo-)R^2 ≥0.25 is considered as significant, a (pseudo-)R^2 between 0.10-0.25 as moderate, and a (pseudo-)R^2 lower than 0.10 as minimal case-mix influence.

Step 7: Fill in the table at the end of this practical guide.

Step 8: Calculate unadjusted and adjusted O/E ratio to calculate RMSE, to determine if case-mix adjustment is necessary.

```
#Model 1: Unadjusted model
#Observed number of events: individual hospital quality score (number of achieved outcomes per hospital)
QI_1 <- QI_1 %>%
  group_by(hospital_id) %>%
  mutate(observed = sum(numerator1  == TRUE))

#Expected number of events: mean from all hospitals for unadjusted model
model_exp_QI_1 <- glm(numerator1  ~ 1,family=binomial,data=QI_1) #define Unadjusted model for mean from all hospitals
QI_1$expected_prob_QI_1 <-
predict.glm(model_exp_QI_1,family=binomial,data=QI_1,type = "response")
#kans per patient

# Calculate expected number of patients per hospital
QI_1 <- QI_1 %>%
  group_by(hospital_id) %>%
  mutate(expected_QI_1 = sum(expected_prob_QI_1))

QI_1a_subset <- QI_1[,c("hospital_id", "observed", "expected_QI_1")]
QI_1a_mod1 <- aggregate(. ~hospital_id, data=QI_1a_subset, FUN=mean)

#O/E rate
QI_1a_mod1$SR_mod1 <- QI_1a_mod1$observed / QI_1a_mod1$expected_QI_1
QI_1a_mod1

#Model 2: case-mix adjusted model
#Observed number of events: similar to model 1

#Expected number of events: predicted probability for an individual hospital for the case-mix corrected model
model_exp_QI_1_2 <- glm(numerator1  ~  case-mix_variables, family = binomial,data = QI_1)

QI_1$expected_prob_QI_1_2 <-  predict.glm(model_exp_QI_1_2,QI_1, type = "response") # predict outcome based on model
```

```
#Calculate expected number of patients per hospital
QI_1 <- QI_1 %>%
  group_by(hospital_id) %>%
  mutate(expected_QI_1_2 = sum(expected_prob_QI_1_2))

QI_1a_2_subset <- QI_1[,c("hospital_id", "observed", "expected_QI_1_2")]

QI_1a_mod2 <- aggregate(. ~hospital_id, data=QI_1a_2_subset, FUN=mean)

#O/E rate
QI_1a_mod2$SR_mod2 <- QI_1a_mod2$observed / QI_1a_mod2$expected_QI_1_2
QI_1a_mod2

QI_1a <- plot(x=QI_1a_mod2$SR_mod2, y=QI_1a_mod1$SR_mod1,abline(coef = c(0,
1)),
             main="QI_1a",
             xlab="Adjusted O/E ratio ",
             ylab="Unadjusted O/E ratio ",
             xlim=c(0,2), ylim=c(0,2))

#Function to calculate RMSE
calculate_rmse <- function(predicted, observed) {
  sqrt(mean((observed - predicted)^2))
}

#Calucalte RMSE
rmse_QI_1a <- calculate_rmse(QI_1a_mod1$SR_mod1, QI_1a_mod2$SR_mod2)
```

Rankability

Rankability reflects the proportion of variation that is not due to chance, and relates to the ability of a QI to make meaningful comparisons between QI scores of individual hospitals. Rankability is calculated using formula [1], in which ρ denotes rankability, τ^2 denotes the variance of a random effects model where hospital is added as a random intercept (i.e., between-hospital variation), and s_i denotes the standard error of the estimated hospital effect for hospital i.

$$\rho = \frac{\tau^2}{\tau^2 + median(s_i{}^2)}$$

As rankability is highly impacted by the number of patients treated per hospital, calculating rankability over a longer period than that is used in practice will overestimate the reliability (as probably more patients will be included per hospital if data period is extended). You can choose to perform a sensitivity analysis in which you extend the study period.

The step-by-step guide proved code for example QI with variable name "QI_1".

Step 1: Install and load packages that are needed.

```
install.packages("readr ")
install.packages("dplyr ")
install.packages("lme4 ")
install.packages("rms ")
library(readr)
library(dplyr)
library(lme4)
library(rms)
```

Step 2: Load your dataset into R. We assume that this is data in which missing baseline characteristics are imputed and that the data contains the right analysis period and all the numerator and denominator variables. Choose the correct way to load your data, depending on file type.

```
data <- read_rds("your_dataset.rds")
```

Step 3: Select patients of each denominator and create separate data frame

```
QI_1 <- subset(data, denominator_QI_1 == TRUE)
```

Step 4: Estimate a logistic regression with hospital id included as a fixed effect for binary QIs. Estimate a linear regression with hospital id included as a fixed effect for continuous QIs.

```
#within hospital variation: median sigma2 from fixed effect logistic
regression
QI_1_fixed <- lrm(numerator1 ~  case-mix_variables +
as.factor(hospital_id), data = QI_1)

#extract median(sigma2)
se_numerator1 <- sqrt(diag(QI_1_fixed$var)) #extract se estimates
se_ids_numerator1 <- se_teller3a[..:..] #select the right rows with hospital
se estimates
se_ids_numerator1

#select se of median hospital and square
mediansigma2_numerator1 <- median(se_ids_numerator1)^2
mediansigma2_numerator1
```

Step 5: Estimate a logistic regression with hospital id included as a random effect for binary QIs. Estimate a linear regression with hospital id included as a random effect for continuous QIs.

```
#extract tau2
QI_1_random <- glmer(numerator1 ~  case-mix_variables + (1|id_fusie), data =
QI_1 , family = binomial, control = glmerControl(optimizer = "bobyqa", optCtrl
= list(maxfun = 100000)))
tau2_QI_1 <- as.numeric(VarCorr(QI_1_random))
tau2_QI_1
```

Step 6: Calculate rankability. You can include multiple QIs and corresponding tau and median sigma values (in the right order) to calculate their rankabilities.

```
outcome_vars <- c("numerator1")
tau_values <- c(tau2_QI_1)
mediansigma2_values <- c(mediansigma2_numerator1)

df_rankability <- data.frame(
  QI = outcome_vars,
  Tau2 = tau_values,
  MedianSigma2 = mediansigma2_values
)

df_rankability$Rho   <-   df_rankability$Tau2   /   (df_rankability$Tau2   +
df_rankability$MedianSigma2)

df_rankability
```

Step 7: Classify rankability into low (<50%), moderate (50%-75%), or high (>75%).

Step 8: Fill in the table at the end of this practical guide.

Table: Overview of quality indicator performance

Quality indicator *(column)*	N patients[1]	Feasibility<br>% data available<br>(hospital range)[2]	Discriminative ability<br>Between hospital variation[3]<br>Median (IQR)	Case-mix<br>(Pseudo)<br>R-squared[4]	Reliability<br>Rankability<br>%

IQR = interquartile range; QI = quality indicator. [1]The number of patients in denominator. [2]Hosital range (minimum-maximum) of percentage data available. [3]This is the QI score expressed as a percentage, except for continuous outcomes (if applicable). [4]For continuous outcomes the R-squared is presented instead of the Pseudo R-squared. The colours indicate poor (orange), moderate (yellow) and good (green) performance on the selected criteria.
