# Supplementary material for: Comprehensive Evaluation of Quality Indicators: Analyzing the Dutch Breast Cancer Audit
Source: Int J Health Policy Manag. 2025 Oct 1;14:8943. doi: 10.34172/ijhpm.8943 (PMC12958241; doi:10.34172/ijhpm.8943)
Supplement: Supplementary file 3 — Sensitivity analysis. This supplementary file contains Figure S3 and Table S4. [file ijhpm-14-8943-s003.pdf]

Article title: Comprehensive Evaluation of Quality Indicators: Analyzing the Dutch Breast Cancer Audit

Journal name: International Journal of Health Policy and Management (IJHPM)

Authors' information: Elfi M. Verheul[1,2]¶*, Margrietha van der Linde[1]¶, Hester F. Lingsma[1], Elvira Vos[3], Sabine Siesling[4,5], Linetta B. Koppert[6], NBCA Consortium[#]

[1]Center for Medical Decision Making, Department of Public Health, Erasmus University Medical Center, Rotterdam, The Netherlands.

[2]Dutch Institute for Clinical Auditing, Leiden, The Netherlands.

[3]Department of Surgery, Rhode Island Hospital, Warren Alpert Medical School of Brown University, Providence, RI, USA.

[4]Department of Research, Netherlands Comprehensive Cancer Organization (IKNL), Utrecht, The Netherlands.

[5]Department of Health Technology and Services Research, Technical Medical Centre, University of Twente, Enschede, The Netherlands.

[6]Department of Surgery, Erasmus MC Cancer Institute, Rotterdam, The Netherlands.

***Correspondence to:** Elfi M. Verheul; Email: e.verheul@erasmusmc.nl

Citation: Verheul EM, van der Linde M, Lingsma HF, et al. Comprehensive evaluation of quality indicators: analyzing the Dutch breast cancer audit. Int J Health Policy Manag. 2025;14:8943. doi:10.34172/ijhpm.8943

#A full list of the collaborators of the NBCA Consortium is provided at the end of the article.

¶ Both authors contributed equally to this paper.

Supplementary file 3. Sensitivity analysis. This supplementary file contains Figure S3 and Table S4.

Figure S3. Barplot showing distribution of QI-5 under four different approaches.

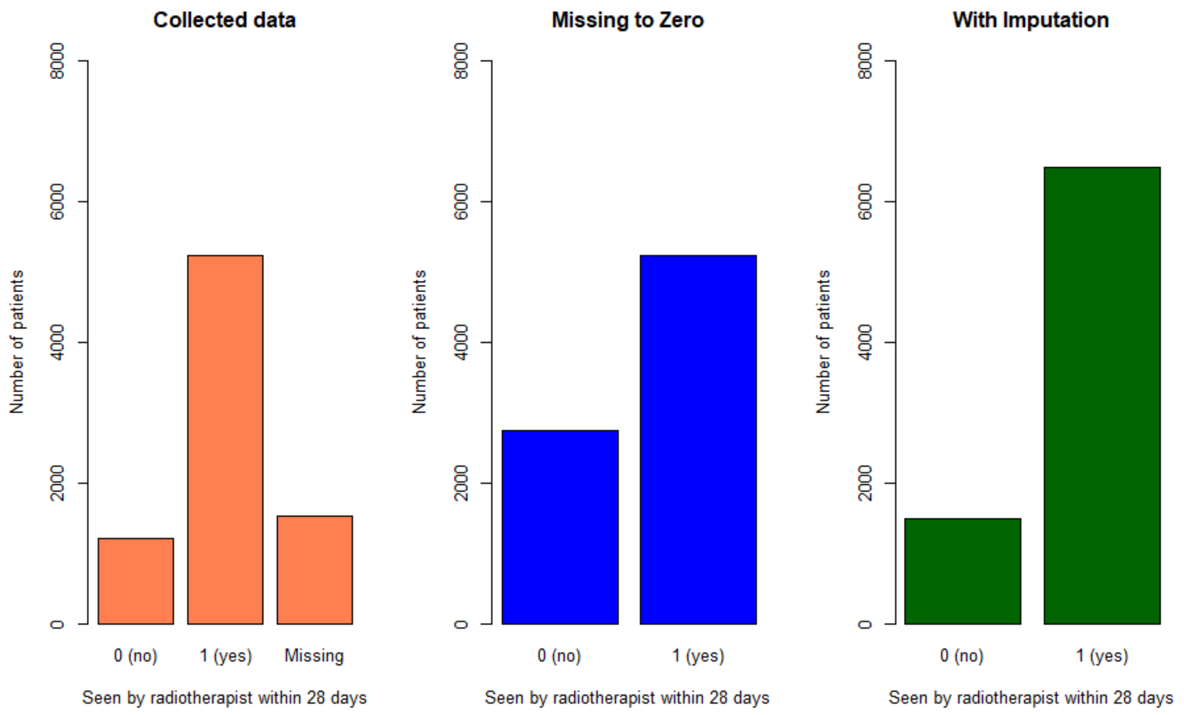

Table S4. Sensitivity analysis of QI-5 using two different approaches for handling missing values: the Missing to zero approach, from our main analysis (also adopted by the NBCA) and the single imputation (using logistic regression) of outcomes of QI-5.

Quality indicator[1]	N patients[2]	Patients per hospital (median, IQR)	Feasibility<br>% data available (hospital range)[3]	Discriminative ability<br>Between hospital variation[4]<br>Median (IQR)	Case-mix<br>(Pseudo)<br>R-squared	Reliability<br>Rankability<br>%
Year(s) of data	**2023**		**2023**	**2023**	**2021-2023**	**2023**
QI-5 (missing to zero, from main analysis)	1812	23 (14-36)	80.2 (0-100)	74 (52-88)	0.02	61
QI-5 with single imputation of outcomes	1812	23 (14-36)	NA	92 (81 – 100)	0.08	72

IQR = interquartile range; QI = quality indicator. *Number of days. [1]See Table 1 for exact definitions of all quality indicators. [2]The number of patients in denominator. [3]Hosital range (minimum-maximum) of percentage data available. [4]This is the QI score expressed as a percentage. The colours indicate poor (orange), moderate (yellow) and good (green) performance on the selected criteria.
